# Sol–Gel Synthesis of NiO-Fe_2_O_3_-SiO_2_/Al_2_O_3_ Catalysts with Statistical and AI-Based Analysis of Experimental Results

**DOI:** 10.3390/molecules30224469

**Published:** 2025-11-19

**Authors:** Aleksandr A. Buzaev, Valerya A. Tkachuk, Konstantin S. Ushenin, Daria N. Staritsyna, Sofya V. Gandybina, Alexandra M. Zakharkiva, Darina K. Ivanova, Ekaterina S. Lyutova, Mariya P. Shcherbakova-Sandu, Irina A. Kurzina, Lyudmila P. Borilo

**Affiliations:** 1 Department of Inorganic Chemistry, Faculty of Chemistry, National Research Tomsk State University, 36 Lenina Avenue, Tomsk 634050, Russia; tk_valeria@bk.ru (V.A.T.); daria.staricina@mail.ru (D.N.S.); sofagand@gmail.com (S.V.G.); alex.zakharkiva@gmail.com (A.M.Z.); darina-ivan-ova-2005@mail.ru (D.K.I.); lyutova.tsu@mail.ru (E.S.L.); mpsandu94@gmail.ru (M.P.S.-S.); kurzina99@mail.ru (I.A.K.); borilo@mail.ru (L.P.B.); 2 Artificial Intelligence for Drug Discovery Center, Presnenskaya Embankment, 6, Building 2, Moscow 123112, Russia; kostanew@gmail.com

**Keywords:** sol–gel synthesis, supported catalysts, NiO, Fe_2_O_3_, catalyst characterization, oxidation reaction, large language models

## Abstract

The development of efficient and cost-effective catalysts is crucial for modern catalytic processes, especially in oxidation reactions. In this study, a sol–gel method was successfully adapted for the synthesis of NiO-Fe_2_O_3_-SiO_2_/Al_2_O_3_ catalysts. The optimized sol–gel process incorporates precise heat treatment control, enabling the production of catalysts with a particle size of 44 nm and a specific surface area of 134.79 m^2^/g. Extensive characterization revealed several significant advantages: a decrease in the heat treatment temperature to 400 °C, maintaining high material dispersion, and eliminating expensive modifiers. Critical synthesis parameters were identified: the Ni/Fe ratio and the heating rate of the heat treatment. Catalytic activity was demonstrated in a model reaction of decane oxidation. Experimental results were confirmed by statistical analysis, and large language models further assisted in the mechanistic interpretation of the results.

## 1. Introduction

In the modern chemical industry, hydrocarbon processing plays a central role in producing a wide range of chemical products and energy carriers. Oxide catalytic systems are crucial in these processes, being widely used in hydrogenation, reduction, and conversion of various organic compounds [1,2].

Traditional catalyst preparation methods based on impregnating supports with metal salt solutions followed by drying and calcination at high temperatures have several significant limitations. The main drawbacks include loss of material dispersion, reduced specific surface area, uneven particle distribution, and coarsening of active components during high-temperature treatment [3]. Additionally, the need to use expensive modifiers such as palladium significantly increases the cost of catalyst production.

The growing demand for efficient and cost-effective catalytic systems in the global market highlights the urgent need to develop new catalyst preparation methods. Sol–gel technology represents a promising approach, allowing the production of materials with controlled structure and properties at relatively low processing temperatures [4,5]. Nickel–iron catalysts currently occupy a significant share (over 35%) in hydrocarbon processing applications [6,7], with an increasing trend towards their use in renewable feedstock processing, including lignocellulosic biomass.

Bimetallic iron–nickel catalysts attract particular research interest due to the synergistic effect of metal interaction. The addition of iron to the nickel structure allows regulating the electronic and redox properties of nickel, significantly increasing system stability compared to monometallic catalysts [8,9]. In methane decomposition, reforming, and Fischer–Tropsch synthesis processes, nickel provides hydrogenation activity while iron enhances thermal stability and modifies carbon deposition pathways.

The active components NiO and Fe_2_O_3_ effectively activate oxygen and decompose hydrocarbon chains [10]. Alumina is traditionally used as a support due to its excellent stabilizing and structure-forming properties. However, high-temperature calcination can lead to the formation of NiAl_2_O_4_ spinel, reducing the reducibility of the nickel phase [11,12].

The sol–gel method offers unique opportunities for controlling the hydrolysis and polycondensation of initial components, ensuring their uniform distribution at the nanoscale [13,14].

Tetraethoxysilane plays a crucial role in forming the catalytic system, acting as a binding agent and ensuring strong adhesion of active components to the Al_2_O_3_ support [15]. Its molecular structure (Si(OC_2_H_5_)_4_) enables effective interaction with both the support surface and catalyst active components. During hydrolysis, silanol groups Si-OH form, creating strong chemical bonds with the support’s hydroxyl groups.

The formation of a silica matrix at relatively low temperatures avoids high-temperature sintering of catalyst particles. The porous structure of the resulting matrix helps preserve the high specific surface area of the catalyst, while the three-dimensional network formed by tetraethoxysilane stabilizes the distribution of active components and prevents their aggregation [16].

An important aspect of developing new catalytic systems is the need to optimize the composition and morphology of the active phase to achieve maximum catalytic activity. Modern research shows that controlling the size and distribution of metal nanoparticles on the support surface, as well as the ratio of nickel to iron in the bimetallic system, can significantly affect the selectivity and stability of the catalyst during long-term operation. The use of advanced characterization methods, including X-ray diffraction and scanning electron microscopy allows for a detailed study of the structural and phase composition of the obtained catalysts, which is necessary for understanding the mechanisms of their operation and further improvement of the synthesis method [17,18].

The aim of this study is to develop a method for obtaining nickel–iron catalytic materials using sol–gel technology, which allows overcoming the limitations of existing methods and obtaining highly efficient catalysts at moderate processing temperatures. The expected result is the determination of synthesis parameters that reduce the negative impact of thermal treatment on the textural properties of catalytic materials.

## 2. Results

**Figure 1:**
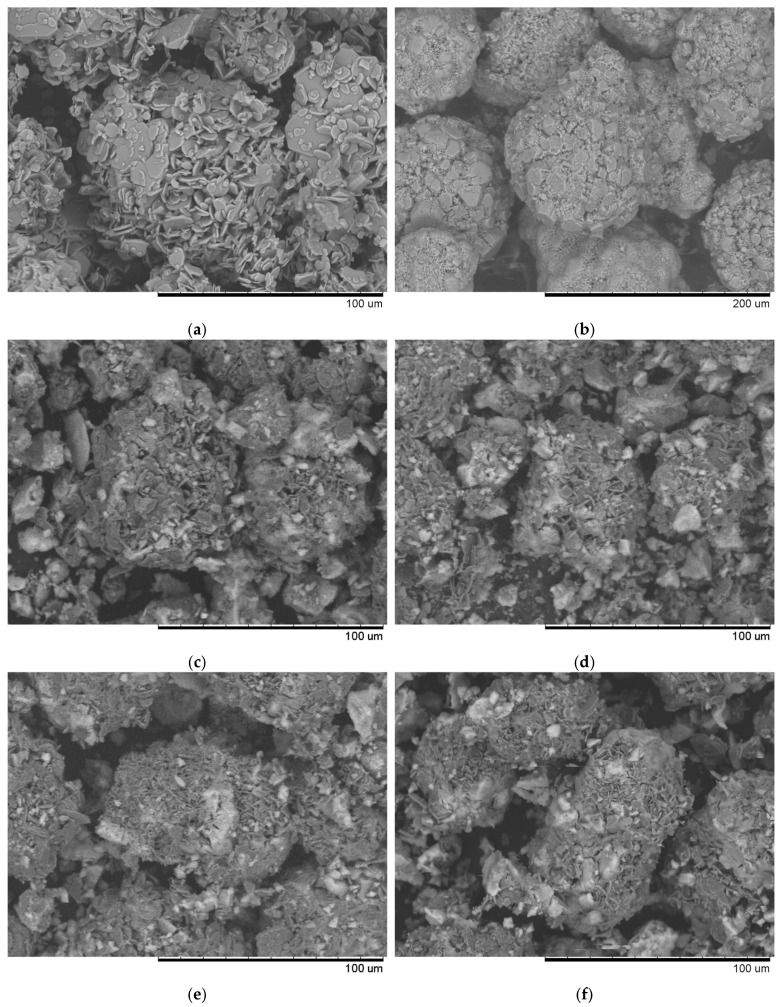
Typical SEM images of the samples obtained at a heating rate of 1 °C/min: (**a**) prepared Al_2_O_3_ support; (**b**) sample with a Ni/Fe ratio of 1/1; (**c**) sample with a Ni/Fe ratio of 1/20; (**d**) sample with a Ni/Fe ratio of 5/15; (**e**) sample with a Ni/Fe ratio of 15/5; (**f**) sample with a Ni/Fe ratio of 20/1.

Visual analysis of SEM images (Figure 1) of synthesized samples with different Ni/Fe ratios at a heating rate of 1 °C/min revealed significant differences in the structure of the studied materials. At a Ni/Fe ratio of 1/1, the formation of homogeneous particles with a solid structure is observed, indicating adhesion of the deposited components to the support surface. For Ni/Fe ratios of 20/1; 15/5; 5/15, and 1/20, fragmentation and disruption of the structure’s integrity are observed, along with the formation of aggregates unbound to the support.

**Figure 2:**
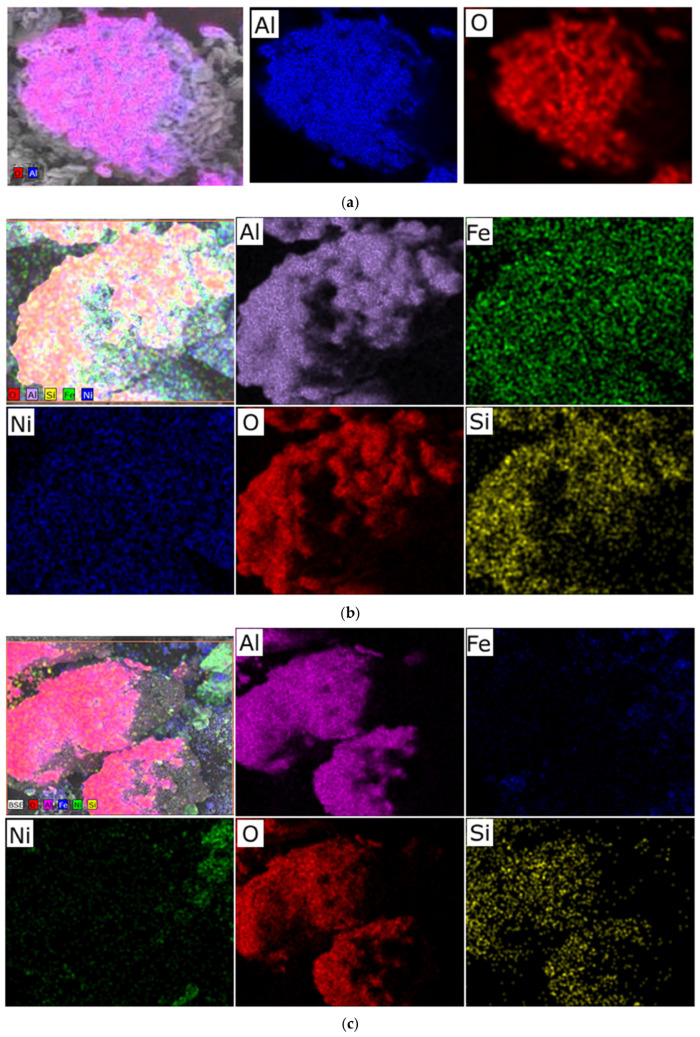
Typical distribution of elements over the sample surface obtained at a heating rate of 1 °C/min: (**a**) prepared Al_2_O_3_ support; (**b**) sample with a Ni/Fe ratio of 1/1; (**c**) sample with a Ni/Fe ratio of 1/20.

**Figure 3:**
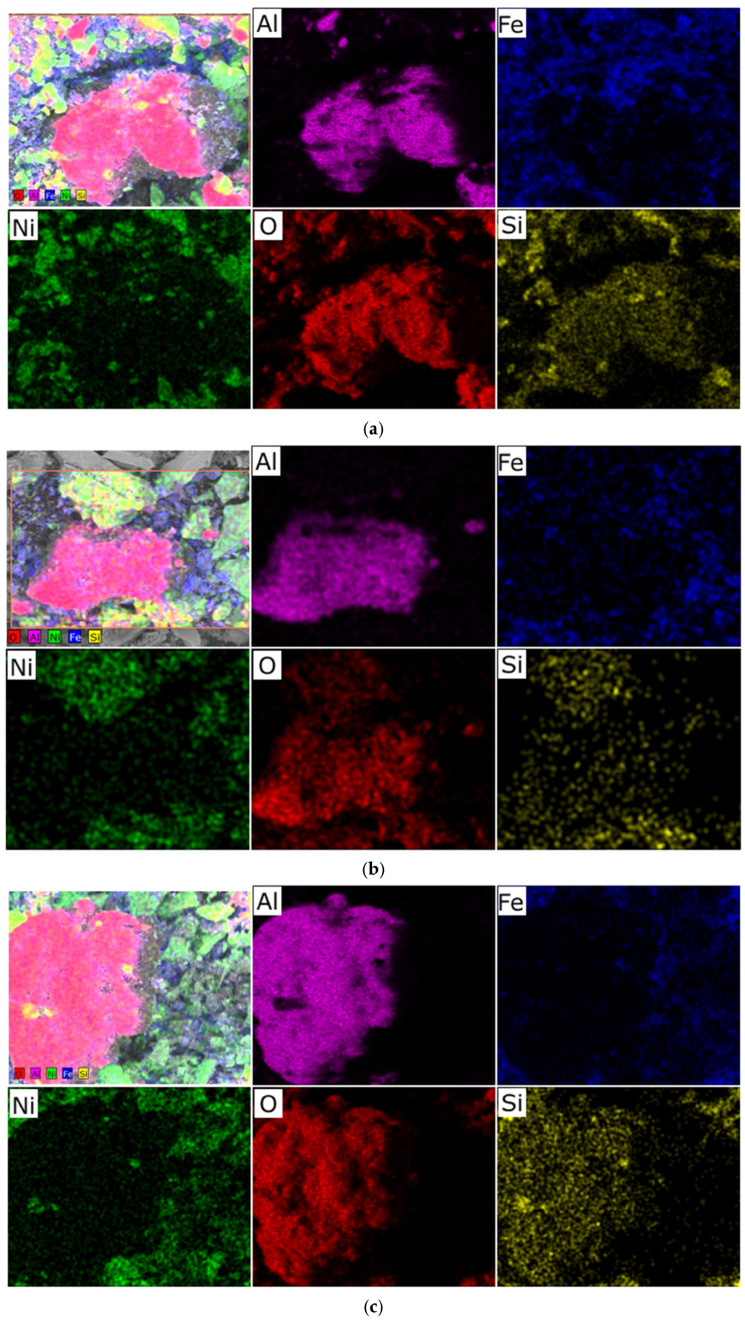
Typical distribution of elements over the sample surface obtained at a heating rate of 1 °C/min: (**a**) sample with a Ni/Fe ratio of 5/15; (**b**) sample with a Ni/Fe ratio of 15/5; (**c**) sample with a Ni/Fe ratio of 20/1.

We hypothesize that an excess of one or the other metal (in the Ni/Fe ratio) leads to a disruption in the uniform distribution of the deposited components and the formation of isolated NiO and Fe_2_O_3_ oxides under the synthesis conditions described in Section 2 of this article, which is confirmed by elemental analysis (Figure 2 and Figure 3).

At a 1/1 Ni/Fe ratio, SEM images reveal a high degree of surface area, with uniformly distributed particles tightly attached to the Al_2_O_3_ substrate. Elemental analysis (Figure 2b) confirms a balanced distribution of nickel and iron across the surface, with no distinct zones of excess of either element, indicating the boundaries of a mixed spinel-type phase. This ensures stable morphology, minimizing aggregation.

In nickel-dominated samples (Ni/Fe = 20/1 and 15/5), SEM images (Figure 1) reveal a fragmented structure with large agglomerates of NiO particles weakly bonded to the substrate, leading to the formation of microcracks. Elemental analysis (Figure 2c and Figure 3) reveals localized zones of high Ni content, while Fe is distributed unevenly, predominantly in the form of isolated clusters. This may be due to kinetic limitations of deposition, where excess Ni precipitates faster, displacing Fe and disrupting integration with Al_2_O_3_. In contrast, iron-dominated samples (Ni/Fe = 5/15 and 1/20) exhibit the formation of porous Fe_2_O_3_ aggregates, characterized by weak adhesion and the presence of voids on the surface. EDS maps (Figure 3) show Fe dominance in the central regions, with minimal Ni distributed along the particle edges. This morphology indicates phase separation, where Fe_2_O_3_ forms distinct domains, potentially reducing catalytic efficiency by reducing the accessibility of active sites. Further detailed analysis focused on the sample with a 1/1 Ni/Fe ratio, as this composition demonstrates the formation of a homogeneous structure with strong adhesion of the active components to the support. This approach will allow the research to remain focused on the most promising 1/1 composition. The results of the study of the other synthesized samples are presented in the “Supplementary Materials” section for interested readers.

**Figure 4:**
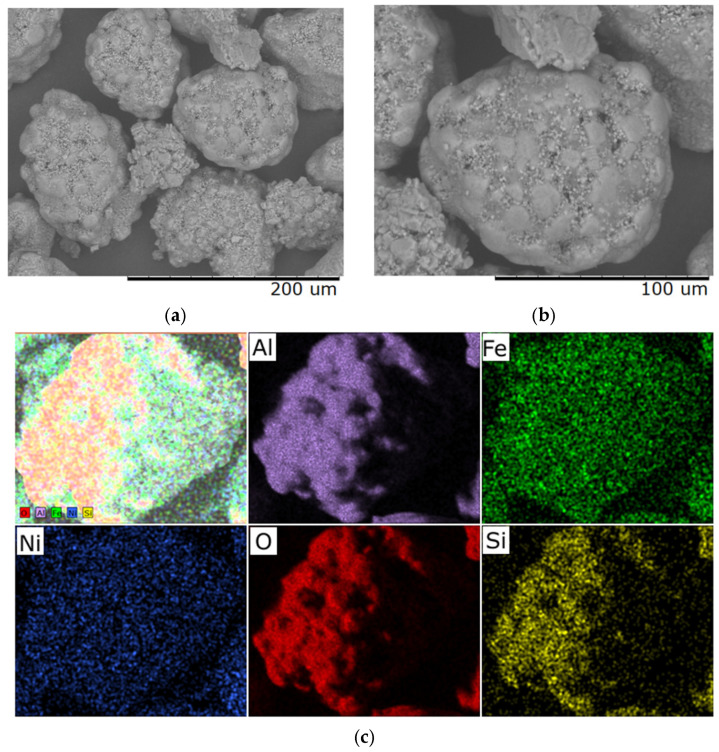
Typical SEM images of samples with a Ni/Fe ratio of 1/1 obtained at a heating rate of 1 °C/min: (**a**) magnification 500×; (**b**) magnification 1000×; (**c**) distribution of elements over the sample surface.

According to the results of scanning electron microscopy and microelement analysis, the heating rate has a significant impact on the morphological and structural characteristics of samples subjected to heat treatment at 400 °C for 40 min. Figure 4 presents SEM images of the sample obtained at a heating rate of 1 °C/min, which exhibits a uniform and smoothed surface relief with reduced porosity.

This structure indicates sufficient time allocated for relaxation of internal stresses during the transition through temperature ranges accompanied by phase transformations. Elemental analysis demonstrates uniform distribution; however, excessive compaction of the material is observed, which may adversely affect its functional properties—for example, reduced activity in catalytic or adsorption processes.

**Figure 5:**
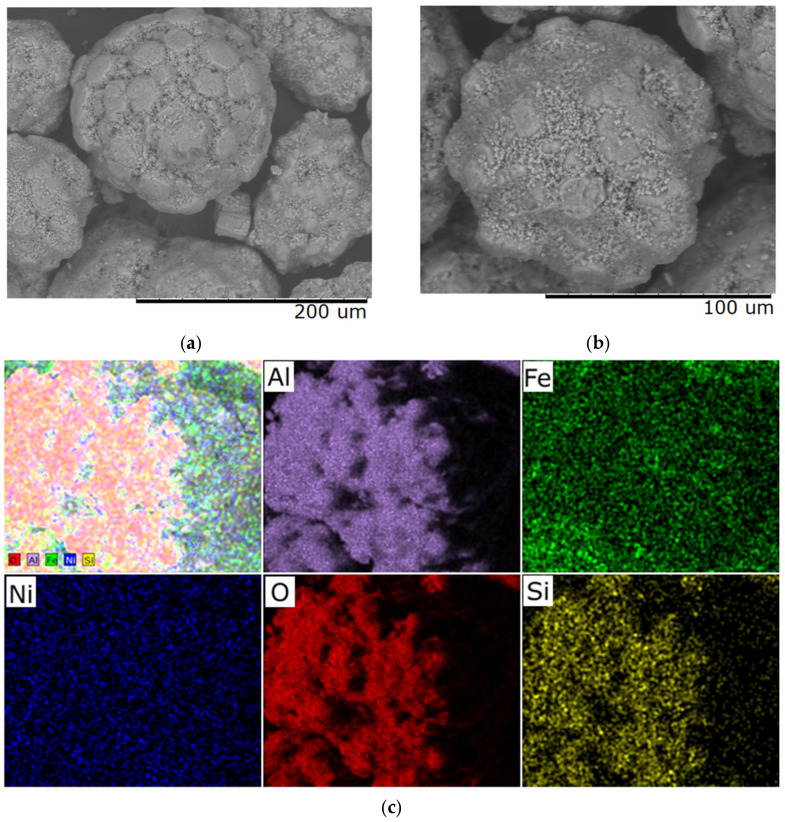
Typical SEM images of samples with a Ni/Fe ratio of 1/1 obtained at a heating rate of 5 °C/min: (**a**) magnification 500×; (**b**) magnification 1000×; (**c**) elemental distribution across the sample surface.

SEM images of samples obtained at a heating rate of 5 °C/min (Figure 5) show that a stable microrelief with distinct textural features has formed, with no signs of cracking. The material surface is characterized by a high degree of homogeneity, which is maintained at the microelement level, indicating structural stability. This heat treatment mode provides a balanced combination of mechanical strength and functional characteristics due to the absence of internal stresses and uniform thermo-diffusion and phase transition processes.

Structural characteristics of the materials obtained at heating rates of 1 °C/min and 5 °C/min demonstrate the formation of dispersed particles. A uniform distribution of the active components Ni and Fe over the surface of the Al_2_O_3_ support is observed. Microscopic studies revealed a lamellar morphology of the agglomerates.

**Figure 6:**
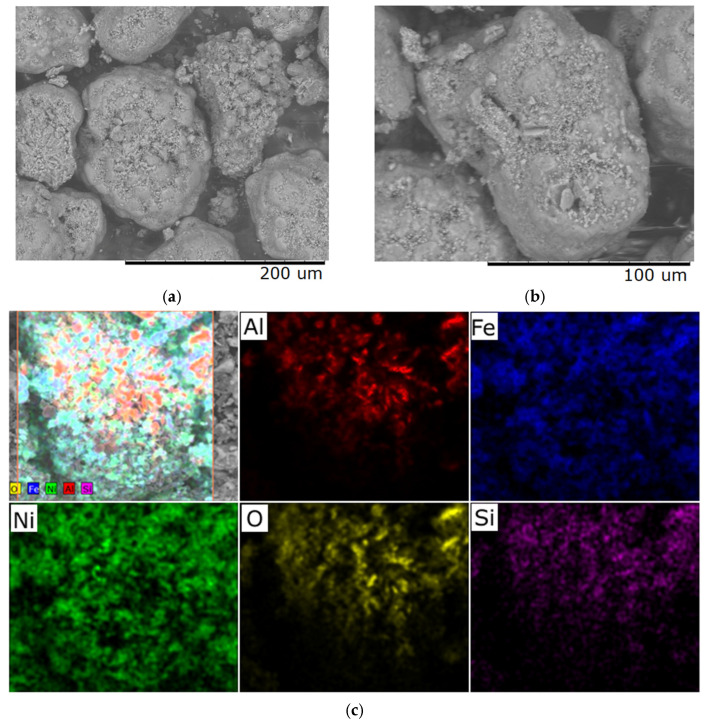
Typical SEM images of samples with a Ni/Fe ratio of 1/1 obtained at a heating rate of 6 °C/min: (**a**) magnification 500×; (**b**) magnification 1000×; (**c**) elemental distribution across the sample surface.

Increasing the heating rate to 6 °C/min leads to the appearance of microcracks, mainly in areas with developed relief, and elemental composition analysis reveals fluctuations in the concentration of individual elements, which may be associated with the onset of phase instability (Figure 6). These changes indicate a violation of the equilibrium conditions for the formation of the material structure as a result of accelerated thermal exposure.

**Figure 7:**
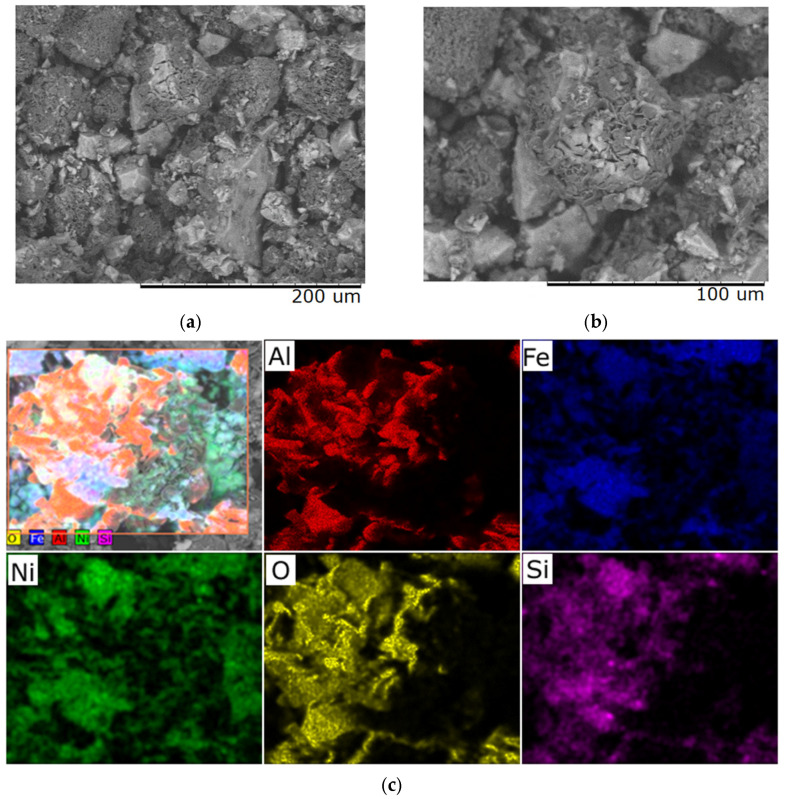
Typical SEM images of samples with a Ni/Fe ratio of 1/1 obtained at a heating rate of 10 °C/min: (**a**) magnification 500×; (**b**) magnification 1000×; (**c**) elemental distribution across the sample surface.

With a further increase in the heating rate to 10 °C/min, the material surface demonstrates pronounced defects in the form of macrocracks, integrity violations, and fragmentation of the upper layer (Figure 7). Microanalysis reveals the presence of zones with altered chemical composition, which is a sign of phase transformations and local structural destabilization. Such changes significantly reduce the quality and functional properties of the obtained material, rendering it unsuitable for most technical applications.

Thus, results were obtained demonstrating the critical influence of heating rate on the morphological parameters and elements in the Supplementary Materials. It was established that only a heating rate of up to 5 °C/min ensures the preservation of the coherent structure and the best possible bond between the catalytically active components and the Al_2_O_3_ surface. Increasing the heating rate above this threshold leads to the occurrence of thermally induced stresses, the formation of microcracks, disruption of the elemental distribution, as well as the destruction of the structural material and the release of catalytically active components from the Al_2_O_3_. These effects are characteristic of all the studied samples, significantly limiting the practical applications of such materials.

**Figure 8:**
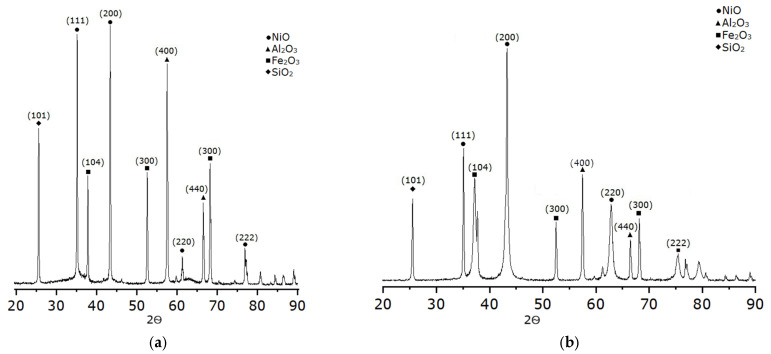
Typical X-ray diffraction patterns of samples with a Ni/Fe ratio of 1/1 (**a**) heating rate 1 °C/min; (**b**) heating rate 5 °C/min.

X-ray diffraction analysis (Figure 8) revealed the following components in the material: nickel oxide (NiO), iron oxides (Fe_2_O_3_), alumina oxide (Al_2_O_3_), and silicon dioxide (SiO_2_). X-ray diffraction patterns of samples heated at 1 and 5 °C/min have similar patterns, with identical phase compositions and peak intensities, regardless of the heating rate. This indicates phase structure stability at low and moderate heating rates, without significant changes in crystallinity or phase interactions.

Analysis of the sample’s diffraction pattern reveals that the main phase is γ-Al_2_O_3_ with characteristic broad reflections (311), (400), and (440), corresponding to the defective spinel portion of the oxide. The presence of high peaks and a lattice parameter (a ≈ 7.94 Å) indicates the preservation of the γ-modification and its stability after the introduction of nickel and iron. The power with γ-Al_2_O_3_ represents an additional phase of NiO (halite-type cubic lattice) and α-Fe_2_O_3_ (hematite), reflecting the partial formation of various metal oxides. The overlap of NiO(111) with γ-Al_2_O_3_(311) confirmed their structural similarity and strong interaction at the interface. The interaction of NiO with γ-Al_2_O_3_ plays a key role in the catalytic activity, as evidenced by the overlap of the NiO(111) and γ-Al_2_O_3_(311) reflections in the diffraction patterns. This indicates a strong bond between nickel and the support, which ensures the preservation of the halite-type cubic structure of NiO. The width of the NiO and Fe_2_O_3_ lines reflects the dispersion and the presence of micro-distortions, confirming a strong bond between the metal and the γ-Al_2_O_3_ support. Weak α-SiO_2_ signals are due to residual silicate inclusions that are not involved in the catalytic activity but affect the textural properties. Thus, the studied sample has a multiphase structure, where γ-Al_2_O_3_ retains the support, while NiO and Fe_2_O_3_ form finely dispersed phases with elements of mutual dissolution and strong interfacial interaction. These characteristics are similar to those of samples at heating rates of 1 and 5 °C/min, including the stability structure in the specified order.

**Figure 9:**
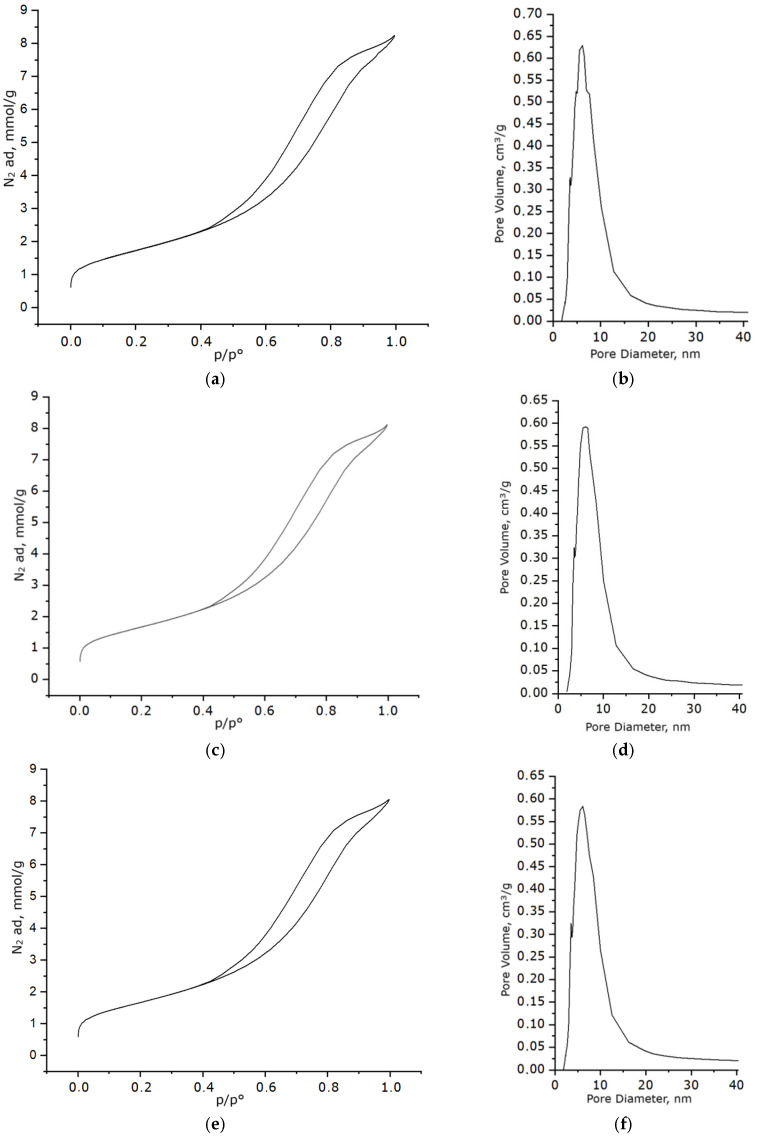
Results of textural studies of the sample: (**a**) nitrogen adsorption–desorption isotherm for the prepared Al_2_O_3_; (**b**) pore size distribution curve for the prepared Al_2_O_3_; (**c**) nitrogen adsorption–desorption isotherm for the sample with a Ni/Fe ratio of 1/1 obtained at a heating rate of 1 °C/min; (**d**) pore size distribution curve for the sample with a Ni/Fe ratio of 1/1 obtained at a heating rate of 1 °C/min; (**e**) nitrogen adsorption–desorption isotherm for the sample with a Ni/Fe ratio of 1/1 obtained at a heating rate of 5 °C/min; (**f**) pore size distribution curve for the sample with a Ni/Fe ratio of 1/1 obtained at a heating rate of 5 °C/min.

The most important parameters determining catalyst efficiency are the specific surface area and porosity of the material. Analysis of the textural characteristics using low-temperature nitrogen adsorption revealed the presence of a developed mesoporous structure in both the support and the synthesized samples. The adsorption–desorption isotherms (Figure 9a,c,e) exhibit a type IV shape with an H3 hysteresis loop, further confirming the mesoporous nature of the material and indicating the possible presence of irregularly shaped pores or pores between aggregated particles. Figure 9b,d,f show the pore size distribution curves constructed based on the desorption branch of the corresponding isotherms. A pronounced maximum is observed in the 2–10 nm range with an average value of 8.32 nm, indicating the dominant contribution of mesopores.

The resulting materials exhibit high specific surface areas. As shown in Table 1, the introduction of the Ni/Fe modification leads to a slight decrease in the specific surface area (by 2.8% at 1 °C/min and by 3.4% at 5 °C/min) and pore volume (by 3.4%) while maintaining a mesoporous structure with a predominant pore size of 8.32 nm. This combination of characteristics indicates a high degree of porosity and a developed surface area, which is important for the catalytic application of the material. The minimal difference between heating rates of 1 °C/min and 5 °C/min further confirms the thermal stability of the porous structure in this range and the absence of significant losses in surface properties.

The textural properties of the samples with Ni/Fe ratio 1/1 on Al_2_O_3_ confirm the stability of morphology at heating rates of 1 °C/min and 5 °C/min, with minor differences. To evaluate the catalytic activity of the obtained materials, model tests were conducted in the decane oxidation reaction at 250 °C and atmospheric pressure. Since the results for the samples with Ni/Fe ratio 1/1, synthesized at heating rates of 1 °C/min and 5 °C/min, were analogous within experimental error, for conciseness of presentation, data are presented only for the sample with a heating rate of 5 °C/min, as this heating rate is a critical value for carrying out the synthesis.

The model catalytic oxidation reaction of decane proceeded mainly via the following reaction scheme:

C_10_H_22_ + 2O_2_ → C_9_H_18_O_2_ + CO + H_2_O + H_2_, (1)

However, due to the multistage nature of the process and the presence of various types of active centers on the catalyst surface, the formation of other oxidation products was unavoidable. During the reaction, the formation of by-products was observed, including carbon dioxide (CO_2_), light hydrocarbons (C_1_–C_8_), heavy hydrocarbons (C_11_ and higher), and carbonyl compounds. The precise identification of light hydrocarbons (C_1_–C_8_) and carbonyl compounds was not performed due to analytical limitations imposed by the close elution times of these components and the nature of the FID detector response.

The comprehensive analysis of product distribution in decane oxidation reaction is presented in Table 2. The table shows molar selectivity’s of all detected products with corresponding measurement uncertainties. The data reflects the results of three runs. One of the main reaction products is C_9_H_18_O_2_ (the target oxygenate), with a selectivity of 38.0 ± 2.0% (RSD = 5.3%). This selectivity level reflects moderate catalyst efficiency but indicates significant side reactions such as complete oxidation. The stoichiometric product CO achieved a selectivity of 35.0 ± 2.0% (RSD = 5.7%), confirming the partial adsorption behavior of the catalyst. Water (H_2_O) and hydrogen (H_2_) were detected with selectivities of 10.0 ± 1.0% (RSD = 10.0%) and 4.0 ± 0.5% (RSD = 12.5%), respectively; however, their accurate quantification is limited by analytical methods (gas chromatography with FID/TCD), and they are partially not quantified due to condensation and adsorption losses.

By-products include CO_2_ (selectivity 5.5 ± 0.7%, RSD = 12.7%), light hydrocarbons C_1_–C_8_ (4.5 ± 0.8%, RSD = 17.8%), and carbonyl compounds (3.0 ± 0.5%, RSD = 15.0%). The high RSDs for these components (up to 17.8%) are due to gas analysis variability and cross-reactions typical of oxidation processes. The overall selectivity of the main products is approximately 88%, and of the by-products is 12%, with a total loss of approximately 3% (untraces or adsorption on the catalyst). The mass balances were 92% for carbon, 88% for hydrogen, and 78% for oxygen, indicating losses likely related to the formation of unaccounted organic traces or oxides on the catalyst surface. These balances are below 100%, which is typical for hydrocarbon oxidation reactions.

**Figure 10:**
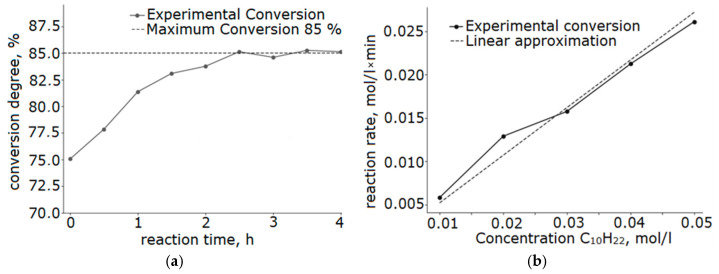
Kinetic curves of the catalytic reaction at 250 °C on a catalyst sample with a Ni/Fe ratio of 1/1 obtained at a heating rate of 5 °C/min: (**a**) decane conversion; (**b**) dependence of the reaction rate on the decane concentration.

Analysis of the kinetic parameters of the reaction revealed a linear dependence of the reaction rate on the concentration of the initial hydrocarbon (Figure 10b). The observed reaction order with respect to decane is close to the first order, which indicates that the process is controlled by the stage of reactant adsorption on the catalyst surface.

**Figure 11:**
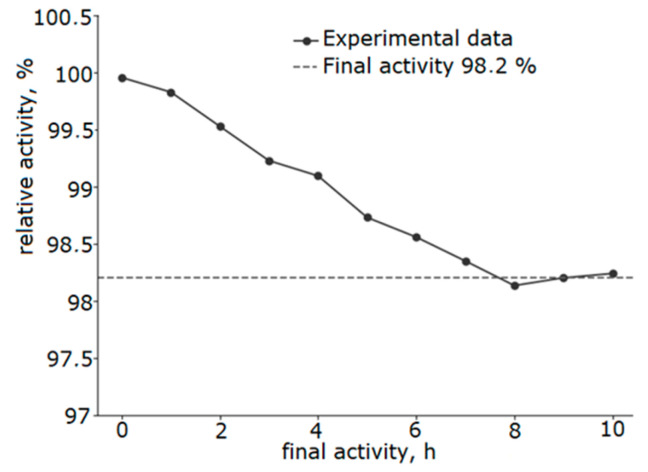
Results of stability evaluation at 250 °C of a catalyst with a Ni/Fe ratio of 1/1 obtained at a heating rate of 5 °C/min.

The catalyst thermal stability was investigated under the conditions of ten-hour testing (Figure 11). After 10 h of operation at a temperature of 250 °C, the catalyst activity decreased by only 1.8%, demonstrating its potential for high stability and promising potential for further industrial study.

Table 3 presents a comparative analysis characteristic of the developed NiO-Fe_2_O_3_-SiO_2_/Al_2_O_3_ catalyst with four analogs from the literature [19,20,21,22]. Specific surface area, measured in m^2^/g, indicates the area of active sites available for reactant adsorption, with higher values enhancing catalytic performance. Particle size, in nanometers, typically improves dispersion and activity when smaller. Processing temperature, in °C, reduces energy costs and prevents particle sintering at lower values. Metal composition indicates active components, where Ni/Fe is bimetallic and cost-effective, while Pd is a noble metal. The analogs are selected for comparison, with all using Ni/Fe except Analog 4 (Pd), highlighting the advantages of the economic composition.

Based on the table data, the developed catalyst demonstrates significant advantages over similar catalysts, confirming the effectiveness of the method used and the optimization of the parameters (Ni/Fe = 1/1, heating rate ≤5 °C/min). The specific surface area of the developed catalyst is 134.79 m^2^/g, which is average among similar catalysts. This surface area provides a sufficient number of active sites for decane oxidation, which correlates with the achieved conversion and selectivity. For particle size, the developed catalyst measures 44 nm, a balanced size for dispersion and stability. This is smaller than Analog 2 (141 nm), indicating superior nanoscale dispersion, as confirmed by SEM data (homogeneous particles without agglomerates). However, it is larger than Analogs 1, 3, and 4 (17–26 nm), which may represent a trade-off for durability, as smaller particles often agglomerate at high temperatures, but the low processing temperature (400 °C) minimizes this risk. The 44 nm particle size is optimal for maintaining porosity (average pore diameter 8.32 nm). The low synthesis temperature (400 °C) and controlled heating rate are among the advantages of the sol–gel method, ensuring stable structure formation without high-temperature sintering. This reduces energy costs and CO_2_ emissions, supporting the principles of “green chemistry”. Compared to Analog 1 (650 °C), where high temperatures can impair dispersion, the developed catalyst preserves the surface after treatment, which may be essential for scaling, as traditional methods require temperatures exceeding 500 °C. For metal composition, the developed catalyst uses Ni/Fe, a bimetallic and economical option, in contrast to Analog 4 (Pd, a noble metal that is expensive and scarce). Ni/Fe provides synergy, with Ni activating O_2_ and C-H bonds, while Fe stabilizes the structure. Analog 3 (Ni, Fe, Co) may offer similar stability, but the developed catalyst excels in temperature and surface area, making it preferable for alkane oxidation.

To verify the stability of the synthesis process and identify statistically significant trends, we analyzed the experimental data using non-parametric and regression-based methods.

**Figure 12:**
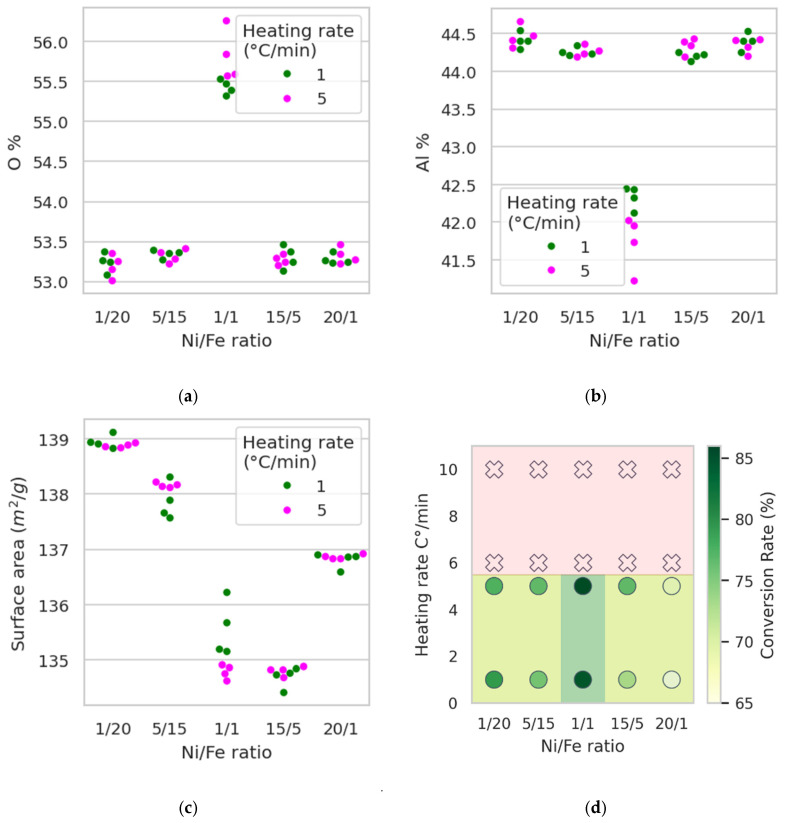
Comprehensive analysis of catalyst performance and structural characteristics: (**a**) percent of Oxygen in samples according to data from scanning electron microscopy; (**b**) percent of Aluminum in samples according to data from scanning electron microscopy; (**c**) dependence of surface area from Ni/Fe ratio and temperature; (**d**) region of the best parameters for synthesis.

As shown above, the Ni/Fe ratio strongly influences grain formation in the catalyst samples, and this trend is confirmed by statistical analysis. In particular, the Al detection in the sample is significantly lower for the Ni/Fe = 1:1 experiment compared to all other tested ratios (20:1, 15:5, 1:20, and 5:15). In contrast, the O content is significantly higher for Ni/Fe = 1:1 than for the other ratios. These results were confirmed using one-tailed Mann–Whitney U tests for each pairwise comparison (five tests for Al and five for O), all of which yielded *p* < 1 × 10^−3^. Figure 12a,b illustrate these effects. Taken together, these results provide statistical confirmation of the qualitative changes in the gelation process at Ni/Fe = 1:1, which is also consistent with the results of visual analysis of the micrographs and indicates the adhesion of the active components to the alumina surface. Temperature exhibits a significant effect on catalyst surface area (*p* = 0.014 < 0.05, one-tailed Mann–Whitney U test for heating rates of 1 °C/min and 5 °C/min). However, the absolute difference in surface area is small for this range of heating rates and is unlikely to be significant for practical applications. Thus, although this effect achieves some statistical significance, its practical significance is low.

To complement the experimental findings, we employed large language models (LLMs) to propose possible mechanistic explanations for the observed trends. The LLMs’ interpretation of the synthesis results is as follows:

- All samples show high surface area (~135–139 m^2^/g), low cracking, and uniform mesoporosity (~8.2–8.3 nm), indicating that: The γ-Al_2_O_3_ support (pre-calcined at 300 °C) provides mechanical stability. TEOS-derived SiO_2_ acts as a mesopore-preserving binder without densifying the network. Nitric acid catalyzes controlled TEOS hydrolysis (Si(OR)_4_ + 4H_2_O → Si(OH)_4_ + 4ROH), preventing rapid gelation and ensuring homogeneous metal incorporation [Qwen-3-Max].

The LLMs suggest the following explanations for the enhanced synthesis performance at a Ni/Fe ratio of 1:1:

- The data clearly shows that the Ni/Fe ratio is the primary factor governing catalytic activity, while the heating rate (1 vs. 5 °C/min) has a secondary effect [DeepSeek V3.2].
- Ni-rich (e.g., 20/1, Samples 17–18): An excess of Ni leads to the formation of larger, segregated NiO nanoparticles. These particles are less interactive with the Fe_2_O_3_, reducing the beneficial redox synergy. Larger NiO crystals are less active and more prone to deactivation (e.g., coking or sintering), explaining the significant drop in Conversion Rate (to ~65–67%) [DeepSeek V3.2].
- Fe-rich (e.g., 1/20, Samples 5–6): An excess of Fe leads to the formation of larger α-Fe_2_O_3_ (hematite) domains. Hematite is known to have lower specific activity and can be less selective than the synergistic mixed phase. While performance is better than the Ni-rich case, it still falls short of the 1/1 benchmark [DeepSeek V3.2].
- Equimolar Ni and Fe nitrate precursors co-hydrolyze and co-condense more uniformly in the TEOS/n-butanol sol, minimizing isolated agglomerates. This yields smaller (<50 nm) and well-distributed NiO–Fe_2_O_3_ nanoparticles on the γ-Al_2_O_3_ surface, consistent with the high surface area (~135 m^2^/g) and narrow pore size (~8.3 nm) [Qwen-3-Max].

The LLMs provide the following insights concerning the effect of the heating rate:

- Performance is nearly identical at 1 and 5 °C/min for Ni:Fe = 1:1 (Conversion 85% vs. 86%; Selectivity 88% vs. 89%) [Qwen3-VL-235B-A22B].
- Kinetic Control Dominates Over Thermal Shock: The slow ramp (1 °C/min) allows gradual solvent removal and hydroxyl condensation, promoting densification without cracking. The faster ramp (5 °C/min) still permits sufficient time for structural relaxation due to the low thermal mass and high porosity of the gel. Both rates avoid rapid decomposition of nitrates that could cause pore collapse or metal segregation [Qwen3-VL-235B-A22B].
- No Significant Sintering Difference: Particle size remains constant (~0.044 µm) across heating rates for 1:1 samples, indicating that the temperature profile does not significantly accelerate Ostwald ripening or grain growth within this narrow window (up to 400 °C) [Qwen3-VL-235B-A22B].

## 3. Discussion

The conducted studies allowed us to identify the optimal synthesis parameters for producing nickel–iron catalytic materials using sol–gel technology, minimizing the negative impact of heat treatment on the textural properties of the catalytic materials.

A Ni/Fe ratio of 1/1 is of particular importance. This ratio ensures the formation of a homogeneous structure with strong adhesion of the active components to the support. Structural homogeneity is confirmed by the uniform distribution of the elements over the catalyst surface and the absence of isolated metal accumulation zones. Deviations from this ratio (either toward a predominance of nickel or iron) lead to a disruption in the uniform distribution of the components and the formation of isolated NiO and Fe_2_O_3_ aggregates. In the case of a predominance of nickel (ratios of 20/1 and 15/5), fragmented structures are formed with large agglomerates of NiO particles weakly bound to the support. Excess iron (ratios of 5/15 and 1/20) leads to the formation of porous Fe_2_O_3_ aggregates with poor adhesion and surface voids. The optimal Ni/Fe ratio of 1/1 ensures the formation of effective interfacial bonds that facilitate the Mars–van Krevelen mechanism.

It has been established that the critical factor is the heating rate—no more than 5 °C/min, which ensures the formation of a stable, defect-free structure. Increasing the heating rate to 10 °C/min leads to the appearance of pronounced defects on the material’s surface, including macrocracks and structural integrity issues. This is because rapid heating prevents sufficient relaxation of internal stresses arising during phase transformations.

At high heating rates, uneven thermal loads are distributed throughout the material’s structure, leading to localized overloads and crack formation. Accelerated thermal exposure disrupts thermal diffusion processes and the uniform distribution of components within the material. The active components (Ni and Fe) and the support (Al_2_O_3_) have different thermal expansion coefficients, and these differences are not compensated for during rapid heating, causing mechanical stress at the phase boundaries and structural failure.

The structural characteristics of the resulting catalyst demonstrate values comparable to those of the original support: a specific surface area of 134.79 m^2^/g, a particle size of 44 nm, and an average pore diameter of 8.32 nm, indicating minimal loss of textural properties under specific thermal processing conditions. Phase analysis confirmed the presence of: NiO, which exhibits high catalytic activity in redox reactions; Fe_2_O_3_ (complements NiO in redox cycles); SiO_2_, which performs a stabilizing function; Al_2_O_3_ serves as a carrier for the active NiO and Fe_2_O_3_ phases, providing a synergistic effect in the Mars-van Krevelen catalytic mechanism. Statistical data processing revealed a linear relationship between the synthesis parameters and the textural characteristics of the material.

The catalytic activity of the material was confirmed in the decane oxidation reaction, where 85% conversion was achieved with a selectivity of 88% for all major products at 250 °C. Material balance analysis revealed high selectivity for the major products (88%) in a multi-stage process. The carbon balance is 92%, the hydrogen balance is 88%, and the oxygen balance is 78%. Carbon deficiency may be due to trace amounts of heavy hydrocarbons, carbon deposits on the catalyst surface, and analytical errors. Hydrogen losses are associated with the formation of gaseous products H_2_ and H_2_O, which are partially carried away with the gas flow. The oxygen balance exhibits the greatest deviation, which is caused by the incorporation of oxygen into surface oxides and the formation of peroxide intermediates during the reaction.

The cost-effectiveness of the synthesis method is confirmed by reducing the processing temperature to 400 °C, taking into account the slow heating rate and eliminating the need for expensive modifiers.

## 4. Materials and Methods

The preparation of the catalytic material involved several sequential stages. The initial stage comprised preparation of a film-forming solution with precise control of component ratios. The following reagents were used as starting materials: 0.2 M nickel nitrate heptahydrate (≥99.0 %, chemically pure), 0.2 M iron(III) nitrate nonahydrate (≥98.5 %, pure for analysis), 0.1 M tetraethoxysilane (≥98.5 %, pure for analysis) as a binding agent, butanol (≥99.8 %, absolute, spectrophotometric grade) as a solvent, and nitric acid (≥99.0 %, chemically pure) for hydrolysis control. Key molar ratios of nickel to iron (Ni/Fe) were varied systematically to investigate their effect on catalytic properties. The following ratios were employed: 1/1, 1/20, 5/15, 15/5, and 20/1. The Ni/Fe ratio was chosen for the following reasons: a 1/1 ratio ensures equal amounts of metals, while 1/20 and 20/1 ratios represent extreme variations where one metal predominates, allowing for an understanding of how each metal functions independently. Intermediate ratios of 5/15 and 15/5 help study the effects of different proportions. This approach allows for the proper arrangement of metals to maximize the efficiency of the catalytic process.

The crystallohydrates of nickel and iron were dissolved in butanol at room temperature with constant stirring for 15 min until complete dissolution. Subsequently, a 0.5 M nitric acid solution was introduced to effectively control hydrolysis processes. The final step of solution preparation involved adding tetraethoxysilane, after which the solution was aged for one hour at room temperature to establish equilibrium in the system and stabilize solution viscosity.

The preparation of the Al_2_O_3_ support received special attention. Prior to applying the stable film-forming solution, the support surface underwent calcination at 300 °C for 30 min. After calcination, the cooled Al_2_O_3_ (≥99.7 %, low-silica grade) sample was immersed in the stable film-forming solution for 30 min with constant stirring.

The key stage of the process was thermal treatment of the obtained system at 400 °C for 40 min. Thermal treatment was conducted at heating rates of 1 °C/min, 5 °C/min, 6 °C/min, and 10 °C/min to determine the critical thermal treatment rate. Cooling of the obtained materials was performed under natural furnace cooling conditions.

The obtained materials were analyzed using a range of modern physicochemical methods. The surface morphology of the samples was examined on a “TM-3000” scanning electron microscope (SEM) (Hitachi, Tokyo, Japan) with an accelerating voltage of 15 kV (electron gun 5 × 10^−2^ Pa and camera for the sample 30–50 Pa). X-ray microanalysis was performed on the “Quanax-70” console (Hitachi, Tokyo, Japan) using energy-dispersive X-ray.

The phase composition of the initial components and the obtained composite materials were determined using a diffractometer “XRD-7000” (Shimadzu, Kyoto, Japan) via CuKα radiation. Survey imaging was performed in the range of reflection angles 2θ = 3–100 with a step size of 0.05°. The accumulation time for each point was 3 s. The phases were decoded and identified using the ICDD diffraction database (PDF-2/Release 2012 RDB).

The porous structure and specific surface area of the samples were studied using nitrogen adsorption at 77 K on an automatic gas adsorption analyzer, “TriStar II” (30–20), manufactured by Micromeritics (Norcross, MN, USA). The specific surface area and pore size distribution of the studied samples were determined based on data obtained from the “3Flex” automated sorption unit, also manufactured by Micromeritics (Norcross, MN, USA), utilizing the Brunauer–Emmett–Teller (BET) method. Before the analysis, the samples were degassed in a vacuum for 2 h at a temperature of 200 °C.

To confirm result reproducibility, a series of catalytic materials was prepared in five replicates. Each sample was analyzed for key physicochemical characteristics, including average particle size, specific surface area, and total material mass.

The evaluation of catalytic activity included measurement of kinetic parameters, determination of reaction rate dependence on reactant concentration, monitoring of long-term stability, and continuous catalyst activity monitoring throughout the experiment. The assessment was performed using a flow catalytic setup equipped with a tubular reactor (8 mm diameter) and 1.8 g of catalyst. The experimental setup was equipped with a Lakeshore Model 336 (Lake Shore Cryotronics, Westerville, OH, USA) temperature controller, an MKS Instruments Baratron Model 627A (MKS Instruments, Andover, MA, USA) pressure sensor, an Agilent 7890B Series (Agilent Technologies, Santa Clara, CA, USA) gas chromatograph with an FID detector, and a Brooks Model 5850E (Brooks Instrument, Hatfield, PA, USA) gas flow controller. The catalyst was pre-activated at 250 °C for 1 h using nitrogen purge. The reactant mixture was fed at 250 °C under atmospheric pressure. During the study, the O_2_/C_10_H_22_ molar ratio was maintained at 2:1, with a 10-hour test duration. Decane conversion was determined through chromatographic analysis using the Agilent 7890B gas chromatograph. The system was configured with a HP-Plot/Al_2_O_3_ capillary column (30 m × 0.32 mm × 5 μm) for separation of hydrocarbons and CO/CO_2_ (FID detector, 250 °C); a DB-WAX capillary column (60 m × 0.25 mm × 0.25 μm) for oxygenates (C_9_H_18_O_2_, aldehydes, ketones) (FID, 220 °C). Calibration was performed using certified standard mixtures (Linde Gas, 1–10 % in N_2_) for CO, CO_2_, and hydrocarbons. Detection limits were CO/CO_2_ < 50 ppm, hydrocarbons < 20 ppm. Gas samples (50 mL) were taken every 30 min via a heated sampling loop (150 °C) to prevent condensation. Liquid products were collected in a cold trap (−20 °C) and analyzed after dilution with hexane.

Decane conversion (X) was calculated using the formula [23,24]:

(2) $$X\;=\;\frac{n_{0}-n}{n_{0}}\cdot 100\%,$$

where $n_{0}$ is the initial amount of decane, and n is the amount of unreacted decane.

Selectivity towards main products was calculated as the ratio of target product quantity to the total amount of formed products [23,24]:

(3) $$S\;=\;\frac{n_{i}\cdot c_{i}}{{\sum (n_{i}\cdot c_{i})}_{all\;products}}\;100\%,$$

where $n_{i}$ is the molar flow rate of product *i*; $c_{i}$ is the number of carbon atoms in product *i*; the sum runs over all detected carbon-containing products.

The carbon balance (CB) was calculated as [23,24]:

(4) $$\mathrm{CB}\;=\;\frac{\sum \left(n_{i}\cdot c_{i}\right)}{n_{C10H22}\cdot 10}\;100\%,$$

where $n_{i}$ is the molar flow rate of product *i*; $c_{i}$ is the number of carbon atoms in product *i*; $n_{C10H22}\cdot 10$ is the total moles of carbon atoms in the feed decane.

Hydrogen balance (HB) was determined as [23,24]:

(5) $$\mathrm{HB}\;=\;\frac{\sum \left(n_{i}\cdot h_{i}\right)}{n_{C10H22}\cdot 22}\;100\%,$$

where $n_{i}$ is the molar flow rate of product *i*; $h_{i}$ is the number of hydrogen atoms in product *i*; $n_{C10H22}\cdot 22$ is the total moles of hydrogen atoms in the feed decane.

Oxygen balance (OB) was calculated as [23,24]:

(6) $$\mathrm{OB}\;=\;\frac{\sum \left(n_{i}\cdot o_{i}\right)}{n_{O2}\cdot 2}\;100\%,$$

where $n_{i}$ is the molar flow rate of oxygen-containing product *i*; $o_{i}$ is the number of oxygen atoms in product *i*; $n_{O2}\cdot 2$ is the total moles of oxygen atoms supplied via feed O_2_.

The chromatograms were processed using Agilent ChemStation (version B.04.03) software, enabling precise determination of peak areas and quantitative content of components in the reaction mixture. The main indicator of stability was the change in catalyst activity over time, which was determined by the dynamics of decane conversion.

Aiming to optimize the parameters of the catalyst, we applied a grid-search experimental design. The first dimension was the heating rate (1, 5, 6, and 10 °C/min). The second dimension was the Ni to Fe ratio (1/20, 5/15, 1/1, 15/5, 20/1). We repeated each synthesis four times and collected data for 80 samples. Then, we selected one sample from each experiment and measured the parameters of the catalyst for 10 samples. We did not perform measurements of conversion rate for poor samples that showed lack of porosity or contained cracks and agglomerations. To analyze the data, we employed non-parametric statistical tests to account for non-Gaussian distributions and fitted a regression model to identify the main factors influencing catalytic performance.

In this study, we use large language models (LLMs) for mechanistic interpretation of results, assuming possible benefits from an additional opinion. Because the methodology for enabling chemical reasoning in LLMs is not yet well established, we design our prompting strategy based on recent approaches proposed in the literature. Specifically, we follow ideas from [25,26]. To use LLMs, we create a large prompt with a precursor list, a step-by-step synthesis procedure, obtained experimental results, and a human opinion about better catalysis. Our prompt uses Markdown format. The table with data includes 10 results without missing values. We provide these results to four LLM chat providers: ChatGPT (version ChatGPT-5.1; version ChatGPT-4o), DeepSeek (version DeepSeek-V3.2), and Qwen (version Qwen-3-VL-235B-A22B; version Qwen-3-Max). All outputs are available in the Supplementary Materials. Here, we present the key opinions of the large language models about the chemistry process. We manually select the deeper ideas that do not look like hallucinations.

## 5. Conclusions

Despite the advances made in the study, certain technological limitations of the developed method must be acknowledged. Key limitations include the heat treatment temperature and the Ni/Fe ratio. Exceeding a heating rate of 6 °C/min leads to a critical deterioration in catalyst quality. This manifests itself in the formation of structural defects and damage to the material’s integrity. The relationship between the active component ratio and catalyst efficiency is particularly noteworthy. Deviations from a Ni/Fe ratio of 1/1 lead to critical structural defects: with an excess of nickel, fragmented structures with large NiO agglomerates are formed, while with a predominance of iron, porous Fe_2_O_3_ aggregates with poor adhesion are formed.

To further improve the synthesis method and expand the practical applications of the developed catalyst, a series of additional studies are planned. For industrial applications, it is particularly important to study the influence of various operational factors on the long-term stability and activity of the catalyst. A detailed analysis of the material’s behavior under varying process parameters, including pressure, gas mixture composition, and temperature fluctuations, is necessary. Scaling up the synthesis technology to pilot plants will allow us to evaluate the reproducibility of the results under industrial conditions and determine the optimal process parameters for larger catalyst batches. Studying the possibility of modifying the catalyst composition with various additives may open avenues for improving its stability during long-term operation and expanding the range of possible applications.

The results obtained during the study are fully consistent with the current objectives and open up broad prospects for the development of technology for creating effective catalytic systems. The proposed method for synthesizing nickel–iron catalysts has potential for application and can serve as the basis for the creation of highly efficient catalysts.

## Figures and Tables

**Table 1:** Comparison of textural parameters of Al_2_O_3_ and Ni/Fe samples with a 1/1 ratio at heating rates of 1 °C/min and 5 °C/min.

Parameter	Al_2_O_3_	Ni/Fe Ratio of 1/1 (a Heating Rate of 1 °C/min)	Ni/Fe Ratio of 1/1 (a Heating Rate of 5 °C/min)
Specific surface area	139.51 m^2^/g	135.55 m^2^/g	134.79 m^2^/g
Particle size	43 nm	44 nm	44 nm
Pore volume	0.29 cm^3^/g	0.28 cm^3^/g	0.28 cm^3^/g
Average pore diameter	8.32 nm	8.32 nm	8.32 nm

**Table 2:** Selectivity and material balance at 85% decane conversion (250 °C, atmospheric pressure) on the catalyst sample with Ni/Fe ratio 1/1 obtained at a heating rate of 5 °C/min.

Component	Molar Selectivity (%)	Relative Std. Dev. (%)	Notes
**Main Products**			
C_9_H_18_O_2_	38.0 ± 2.0	5.3	Target oxygenate
CO	35.0 ± 2.0	5.7	Stoichiometric product
H_2_O	10.0 ± 1.0	10.0	
H_2_	4.0 ± 0.5	12.5	
**By-Products**			
CO_2_	5.5 ± 0.7	12.7	Low level but present; indicated as controlled oxidation
Light hydrocarbons (C_1_–C_8_)	4.5 ± 0.8	17.8	High variability due to cross-reactions
Carbonyl compounds	3.0 ± 0.5	15.0	Oxygen-containing; structure not determined due to analysis
**Material Balance**			
Carbon balance	92.0%		Minor losses (unaccounted for organic traces or adsorption).
Hydrogen balance	88.0%		Losses of H_2_/H_2_O
Oxygen balance	78.0%		Surface oxides/intermediates

**Table 3:** Comparative characteristics of the developed catalyst and analogs.

Parameter	Developed Catalyst	Analog 1 [19]	Analog 2 [20]	Analog 3 [21]	Analog 4 [22]
Specific surface area	134.79 m^2^/g	118 m^2^/g	150 m^2^/g	72 m^2^/g	92.70 m^2^/g
Particle size	44 nm	17 nm	141 nm	26 nm	20 nm
Processing temperature	400 °C	650 °C	500 °C	600 °C	500 °C
Metal	Ni/Fe	Ni/Fe	Ni/Fe	Ni/Fe/Co	Pd

## Data Availability

All data from this study are available as «Supplementary Materials».

## Supplementary Materials

The following supporting information can be downloaded at: https://www.mdpi.com/article/10.3390/molecules30224469/s1.
