# Supplementary figures and images for: Sol–Gel Synthesis of NiO-Fe2O3-SiO2/Al2O3 Catalysts with Statistical and AI-Based Analysis of Experimental Results
Source: Molecules. 2025 Nov 19;30(22):4469. doi: 10.3390/molecules30224469 (PMC12655306; doi:10.3390/molecules30224469)

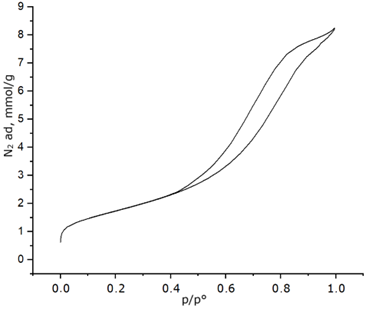

Supplement: Supplementary file 1 [file molecules-30-04469-s001.zip › BET/1a.png]

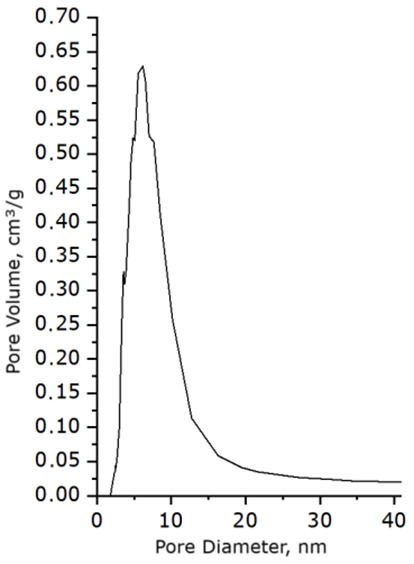

Supplement: Supplementary file 1 [file molecules-30-04469-s001.zip › BET/1b.png]

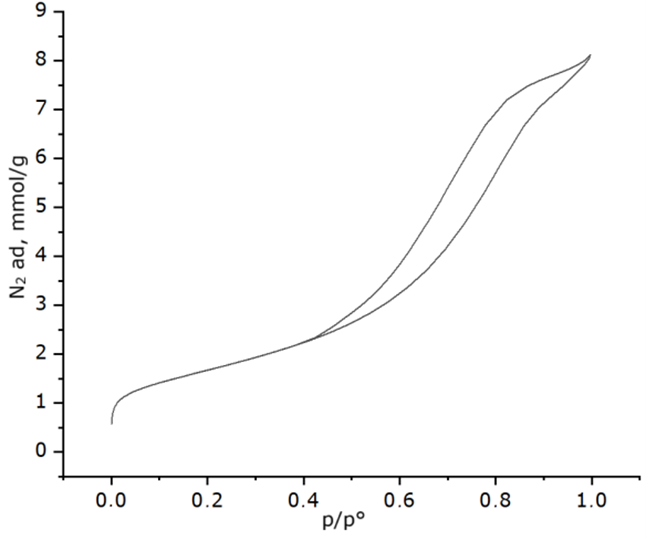

Supplement: Supplementary file 1 [file molecules-30-04469-s001.zip › BET/1c.png]

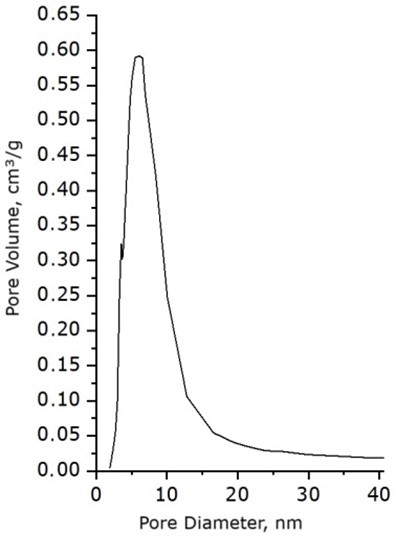

Supplement: Supplementary file 1 [file molecules-30-04469-s001.zip › BET/1d.png]

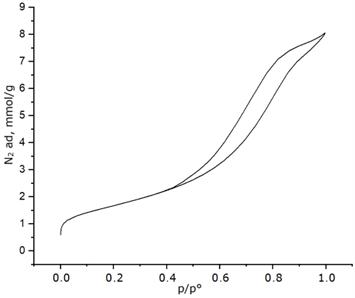

Supplement: Supplementary file 1 [file molecules-30-04469-s001.zip › BET/1e.png]

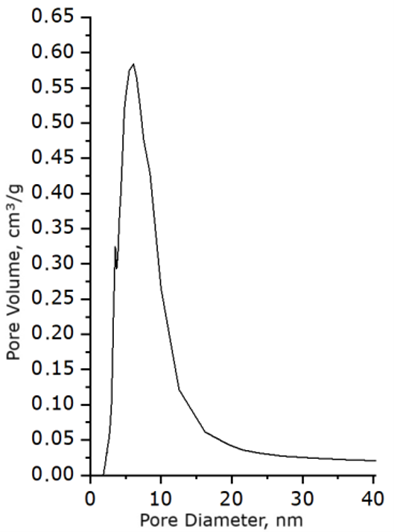

Supplement: Supplementary file 1 [file molecules-30-04469-s001.zip › BET/1f.png]

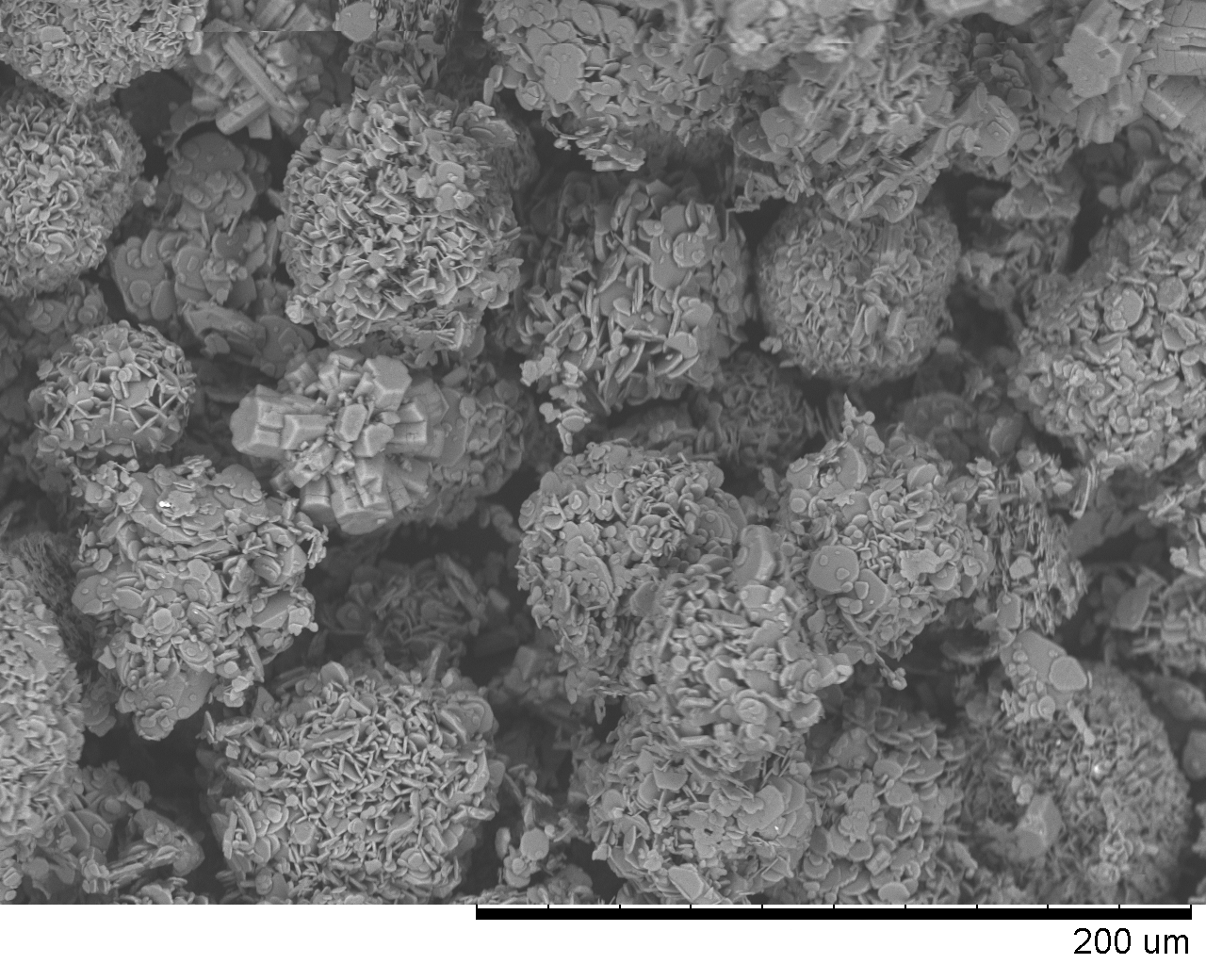

Supplement: Supplementary file 1 [file molecules-30-04469-s001.zip › SEM/Figure 1/prepared Al2O3 support _1.tif]

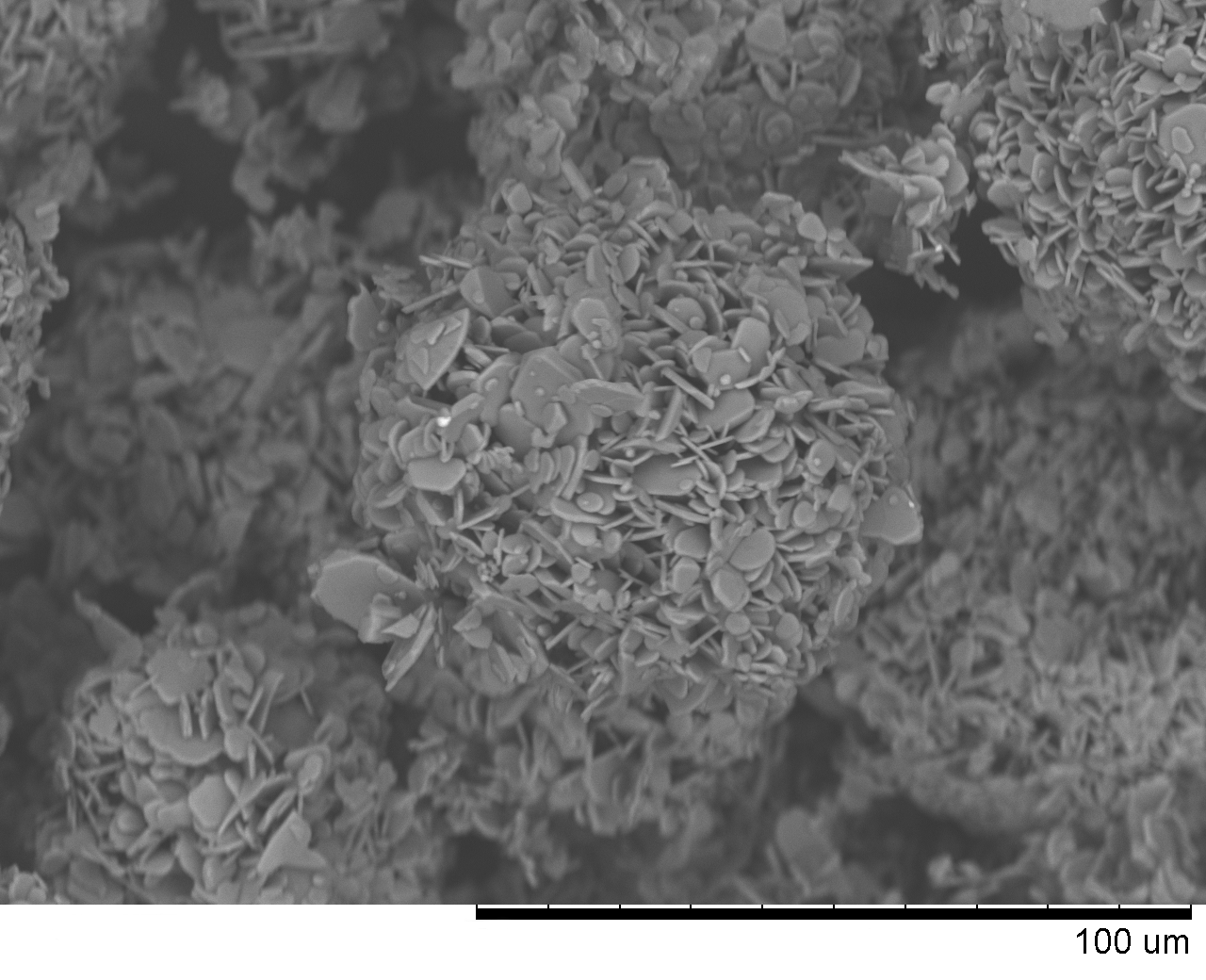

Supplement: Supplementary file 1 [file molecules-30-04469-s001.zip › SEM/Figure 1/prepared Al2O3 support_2.tif]

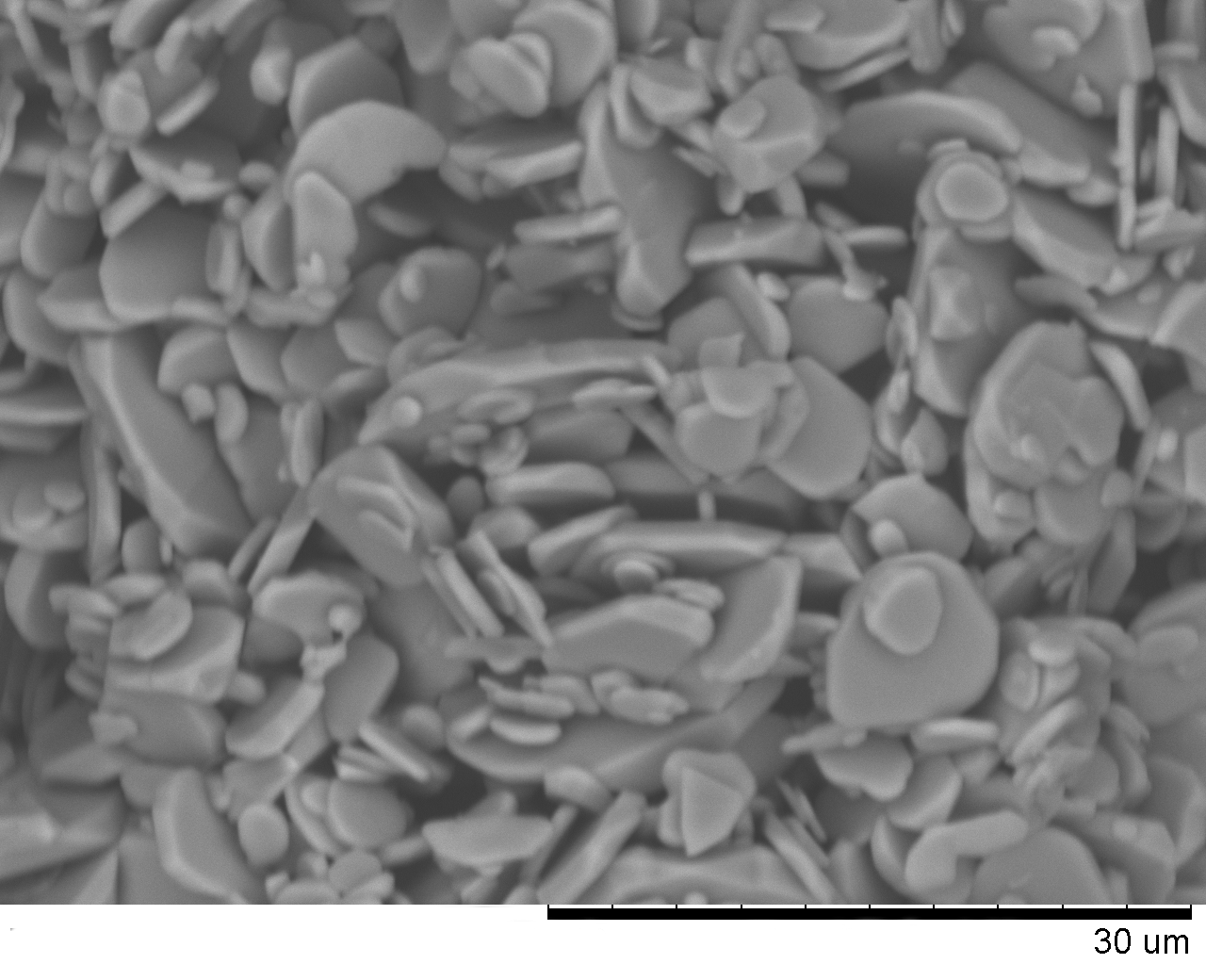

Supplement: Supplementary file 1 [file molecules-30-04469-s001.zip › SEM/Figure 1/prepared Al2O3 support_3.tif]

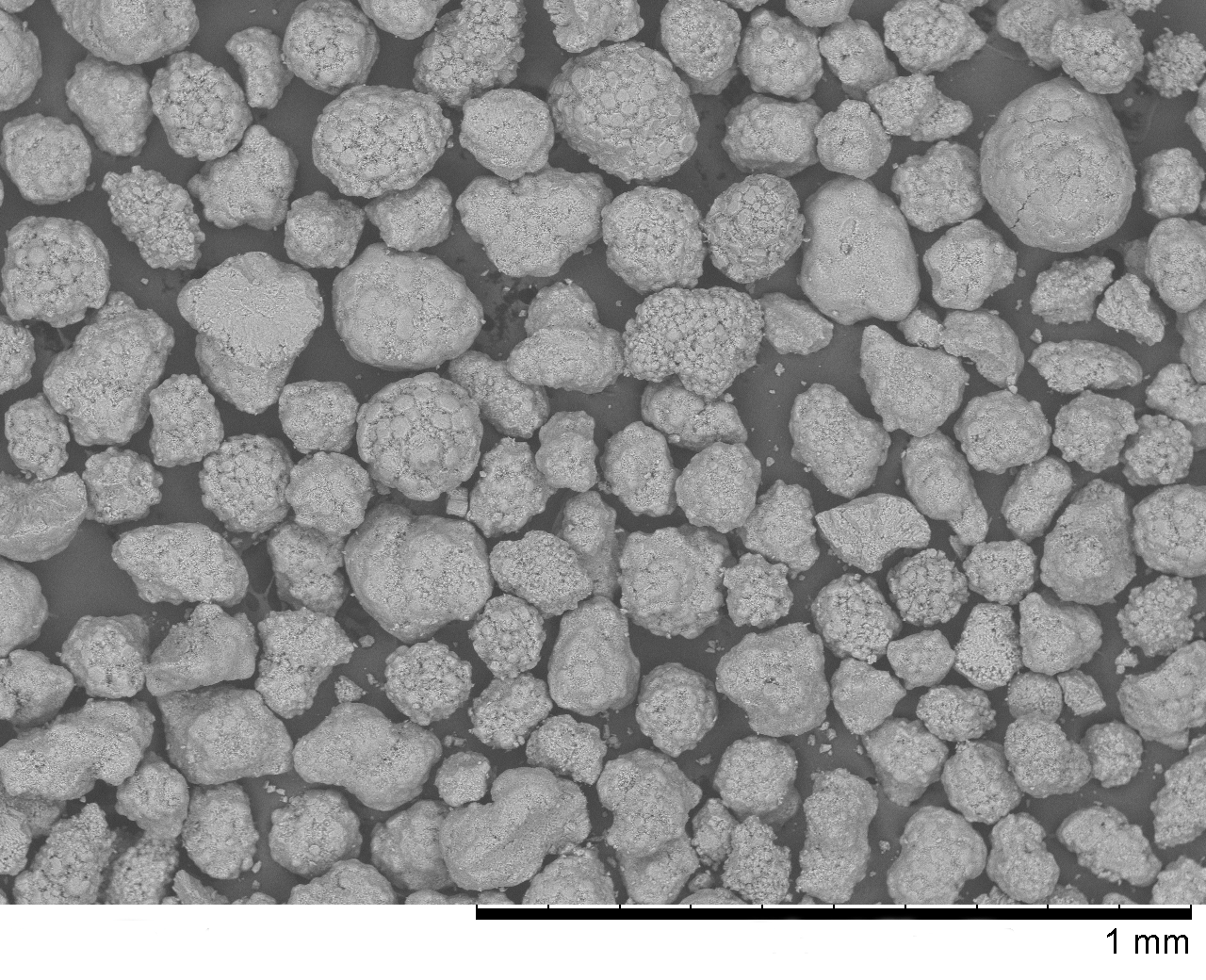

Supplement: Supplementary file 1 [file molecules-30-04469-s001.zip › SEM/Figure 1/sample with a NiFe ratio of 1-1_1.tif]

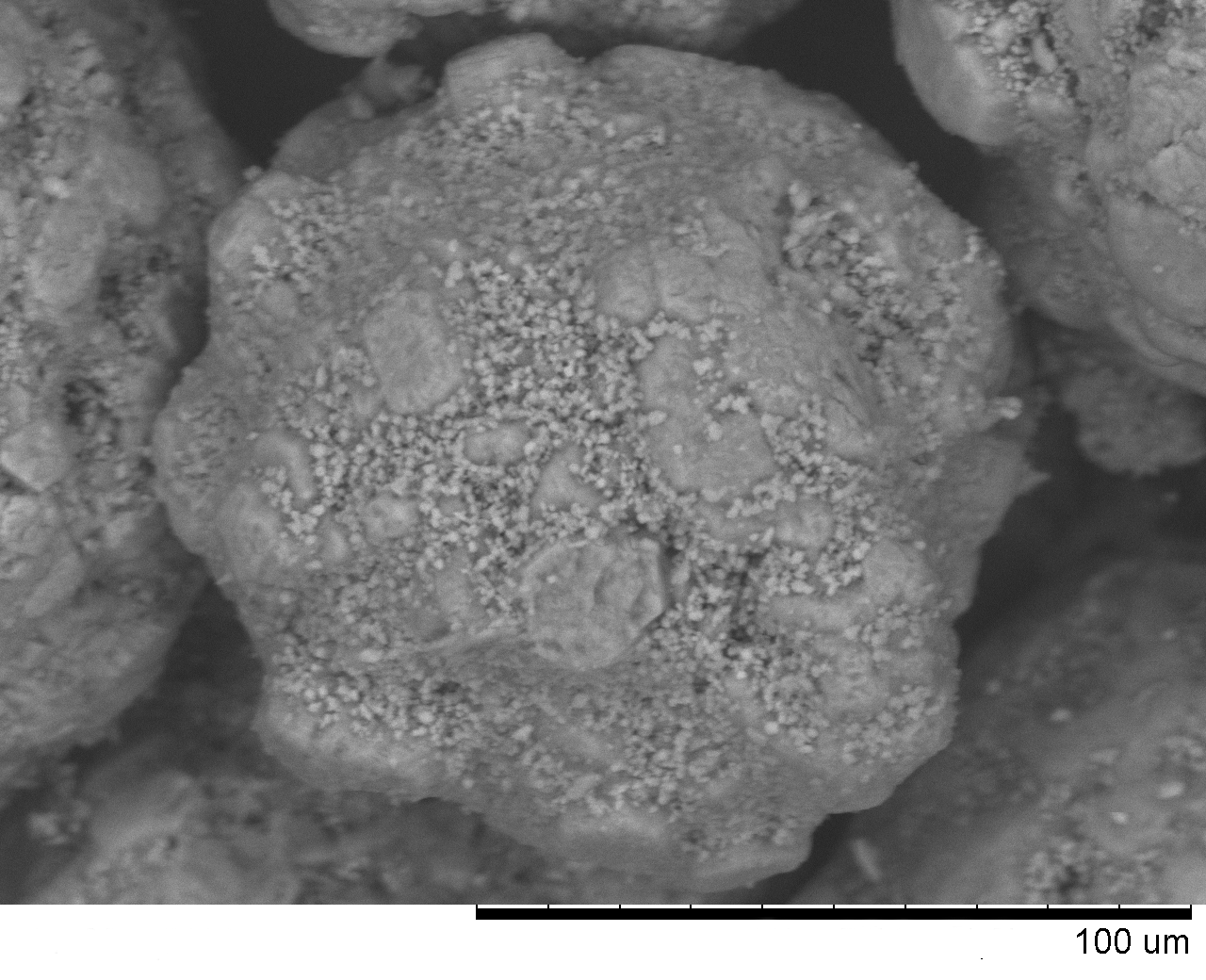

Supplement: Supplementary file 1 [file molecules-30-04469-s001.zip › SEM/Figure 1/sample with a NiFe ratio of 1-1_2.tif]

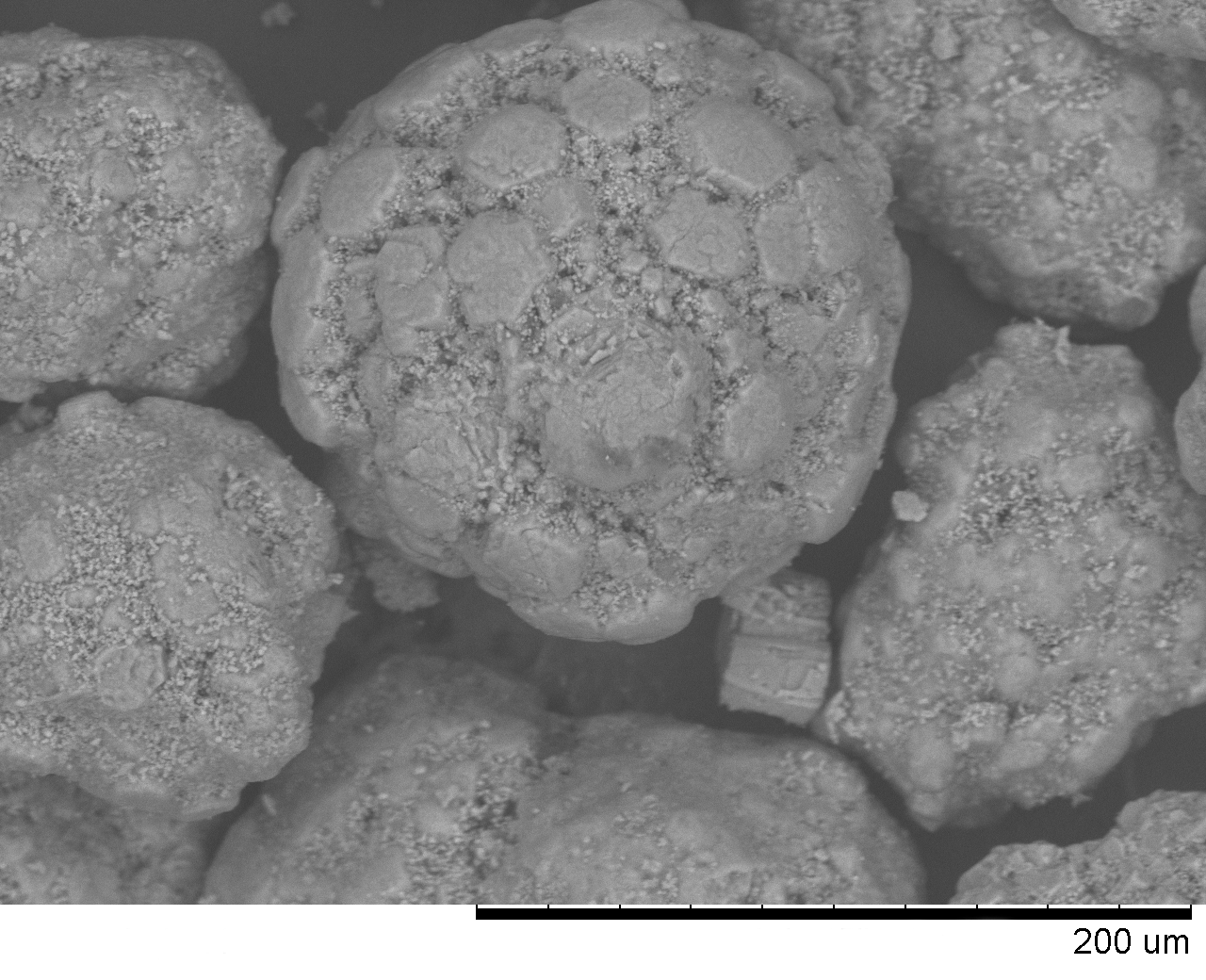

Supplement: Supplementary file 1 [file molecules-30-04469-s001.zip › SEM/Figure 1/sample with a NiFe ratio of 1-1_3.tif]

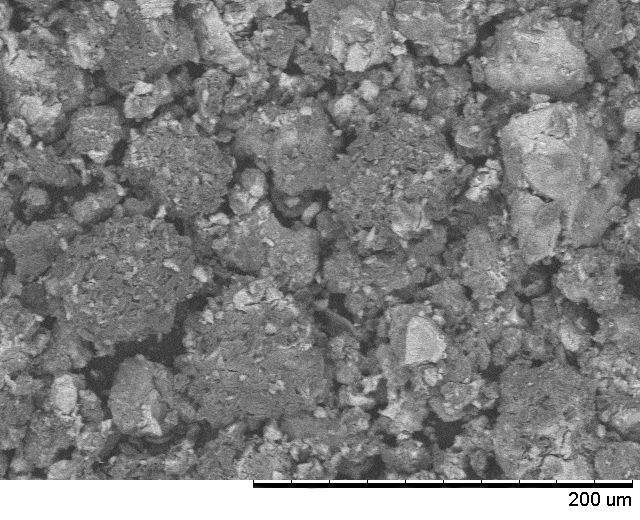

Supplement: Supplementary file 1 [file molecules-30-04469-s001.zip › SEM/Figure 1/sample with a NiFe ratio of 1-20_1.tif]

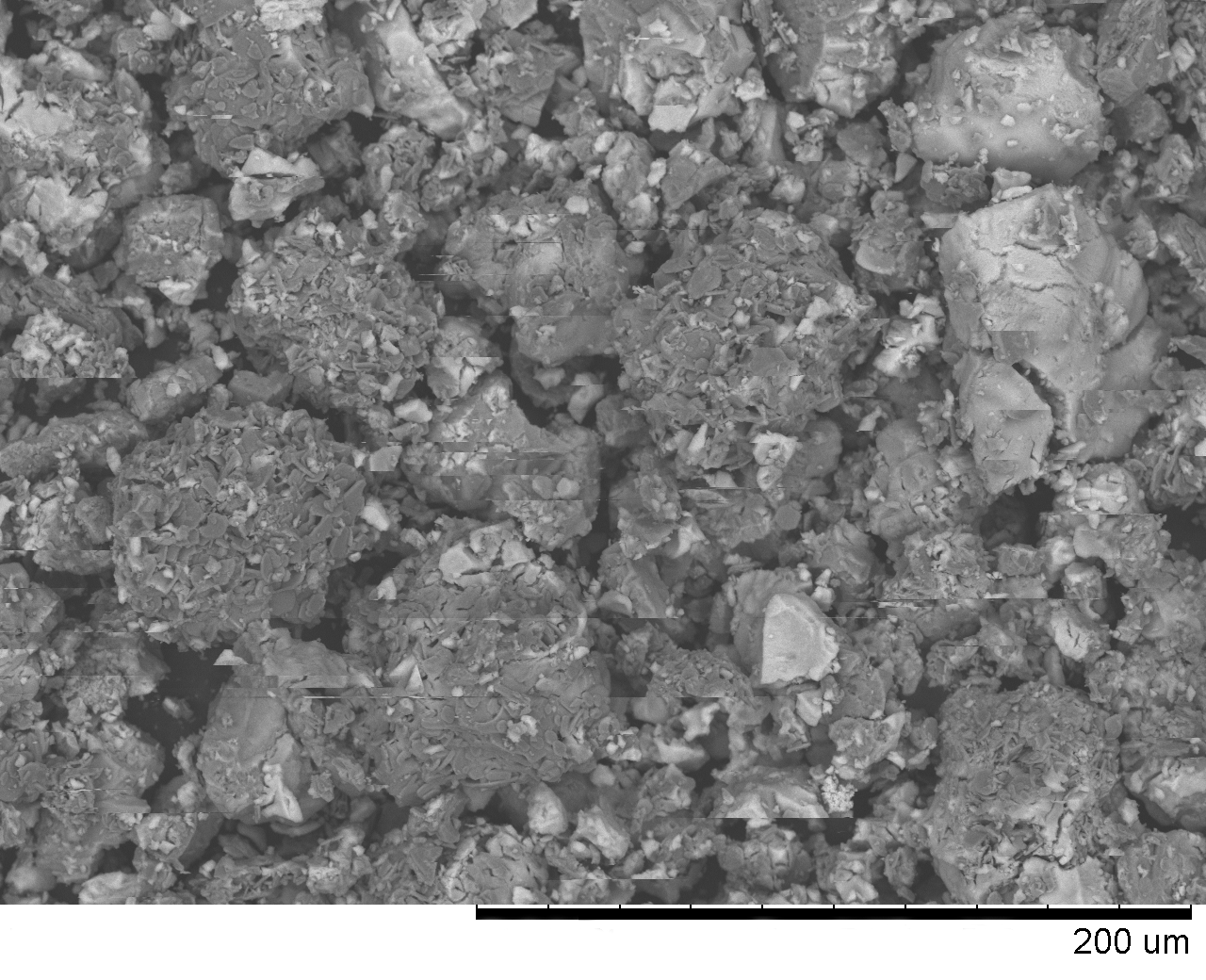

Supplement: Supplementary file 1 [file molecules-30-04469-s001.zip › SEM/Figure 1/sample with a NiFe ratio of 1-20_2.tif]

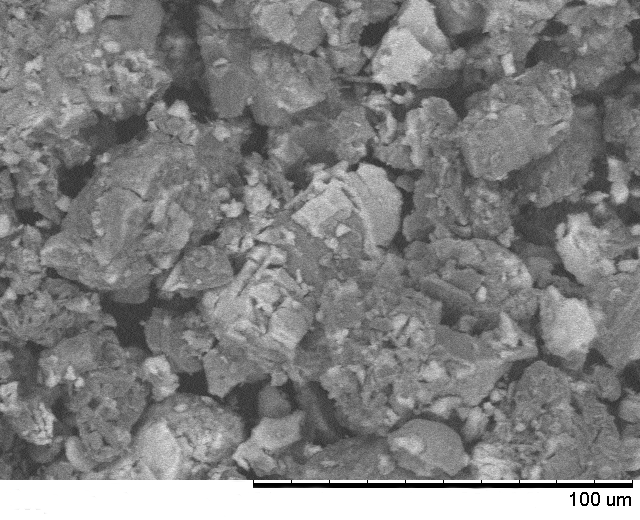

Supplement: Supplementary file 1 [file molecules-30-04469-s001.zip › SEM/Figure 1/sample with a NiFe ratio of 1-20_3.tif]

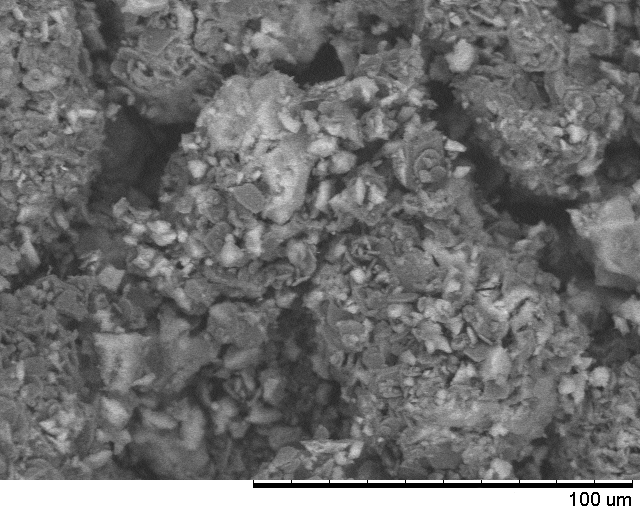

Supplement: Supplementary file 1 [file molecules-30-04469-s001.zip › SEM/Figure 1/sample with a NiFe ratio of 15-5_1.tif]

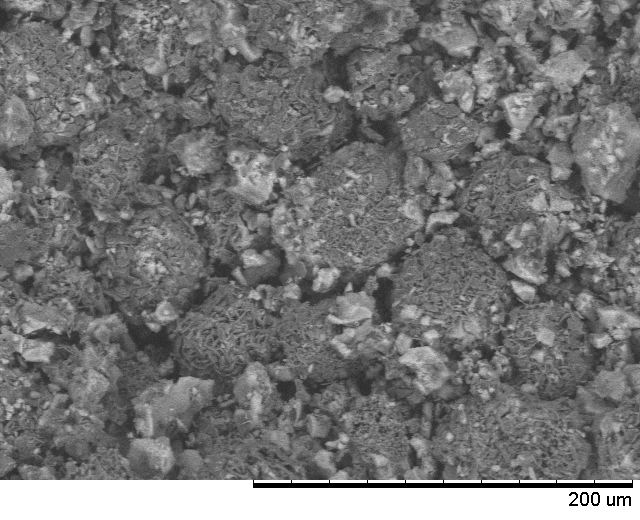

Supplement: Supplementary file 1 [file molecules-30-04469-s001.zip › SEM/Figure 1/sample with a NiFe ratio of 15-5_2.tif]

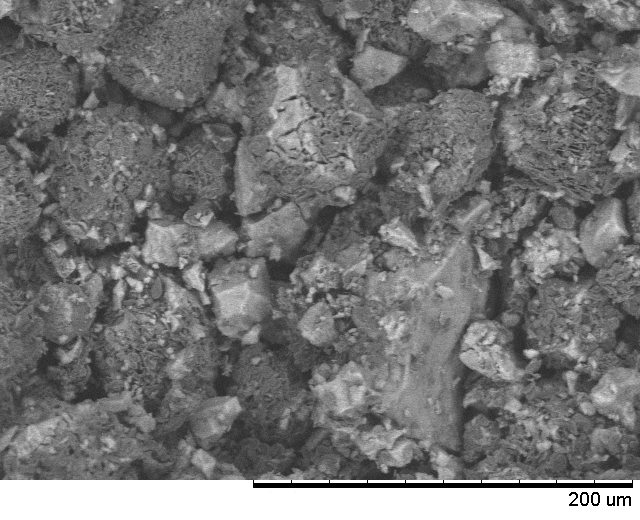

Supplement: Supplementary file 1 [file molecules-30-04469-s001.zip › SEM/Figure 1/sample with a NiFe ratio of 15-5_3.tif]

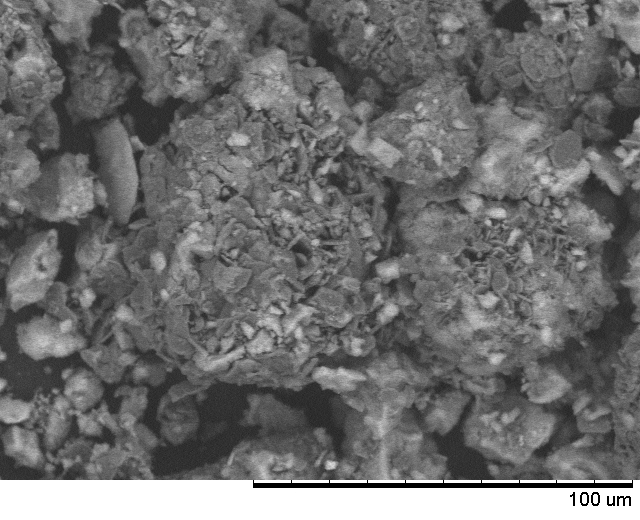

Supplement: Supplementary file 1 [file molecules-30-04469-s001.zip › SEM/Figure 1/sample with a NiFe ratio of 20-1_1.tif]

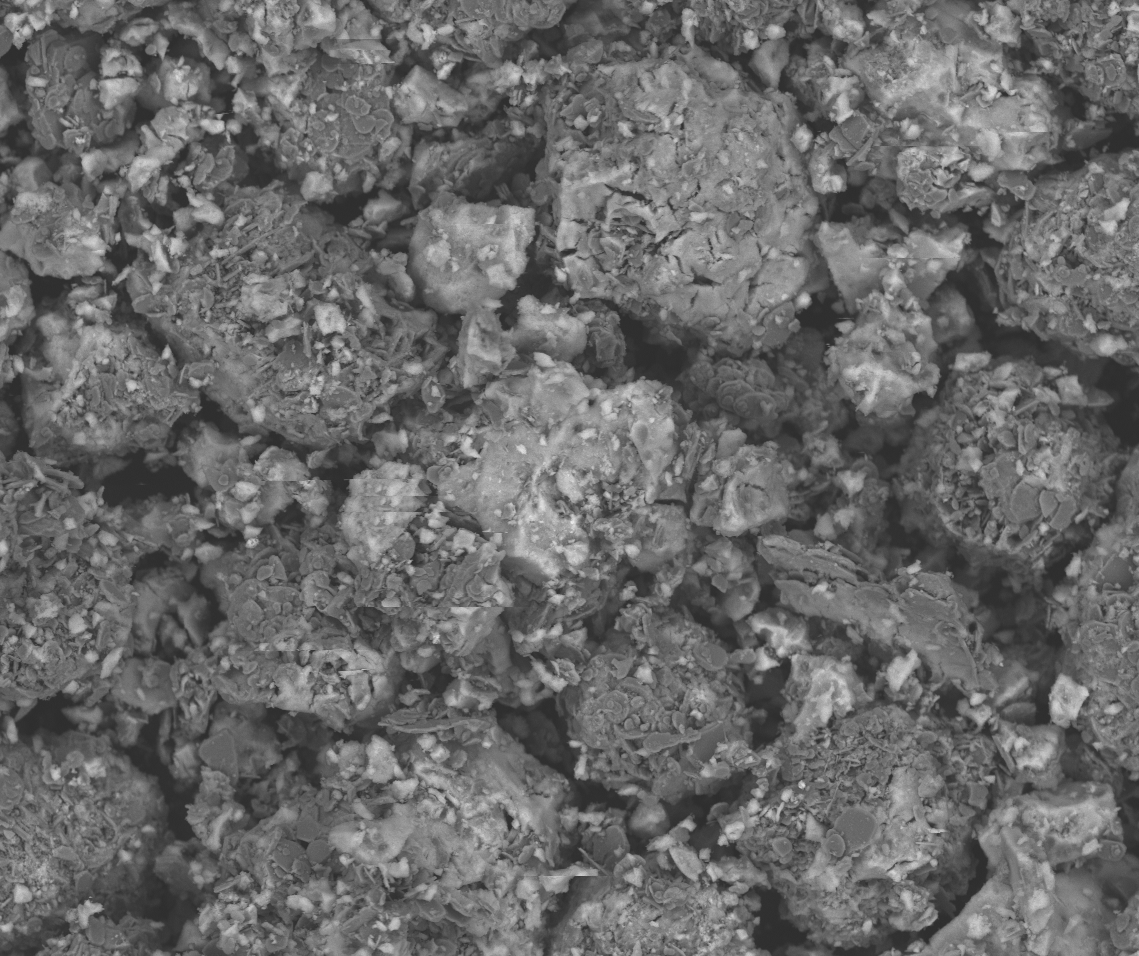

Supplement: Supplementary file 1 [file molecules-30-04469-s001.zip › SEM/Figure 1/sample with a NiFe ratio of 20-1_2.tif]

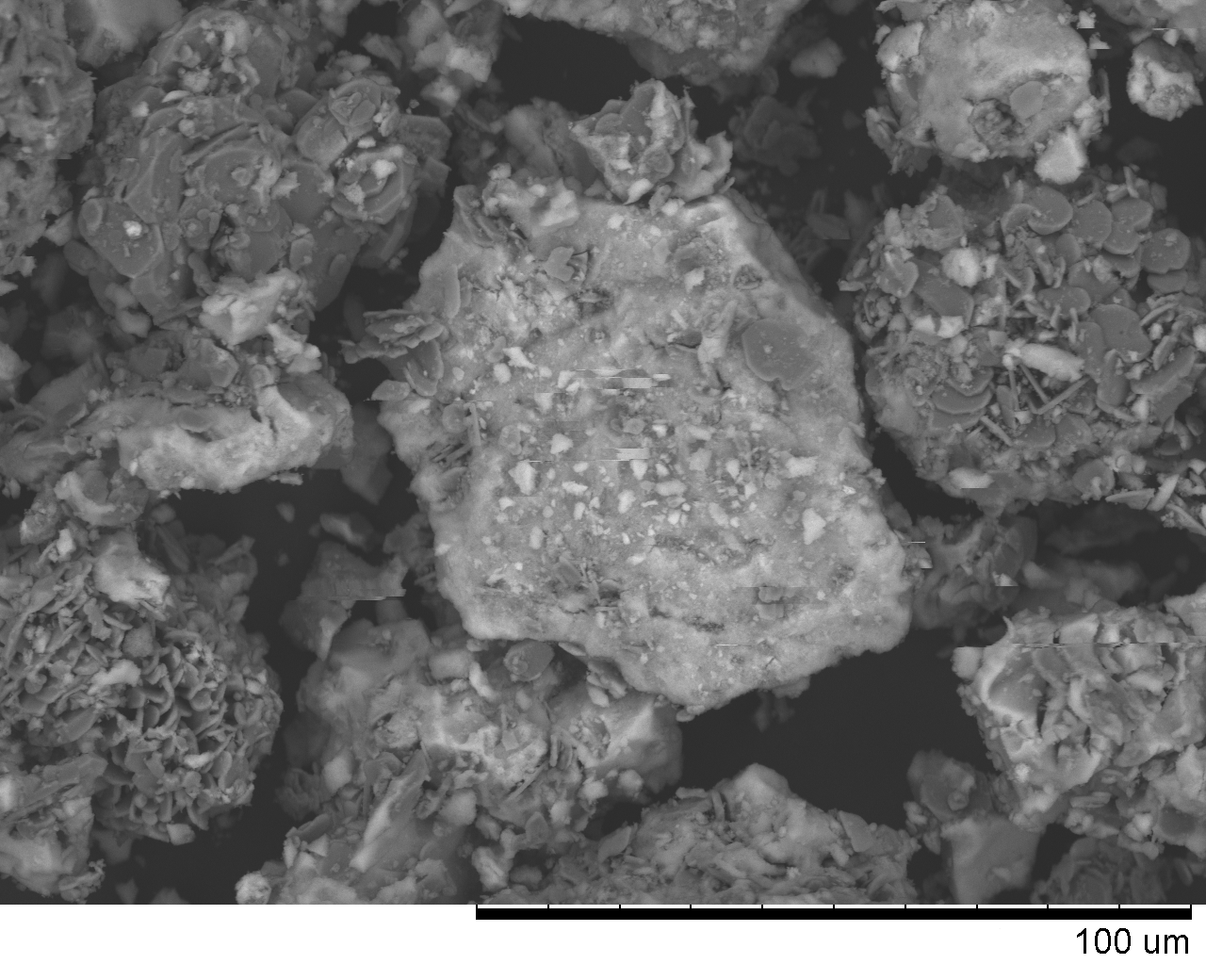

Supplement: Supplementary file 1 [file molecules-30-04469-s001.zip › SEM/Figure 1/sample with a NiFe ratio of 20-1_3.tif]

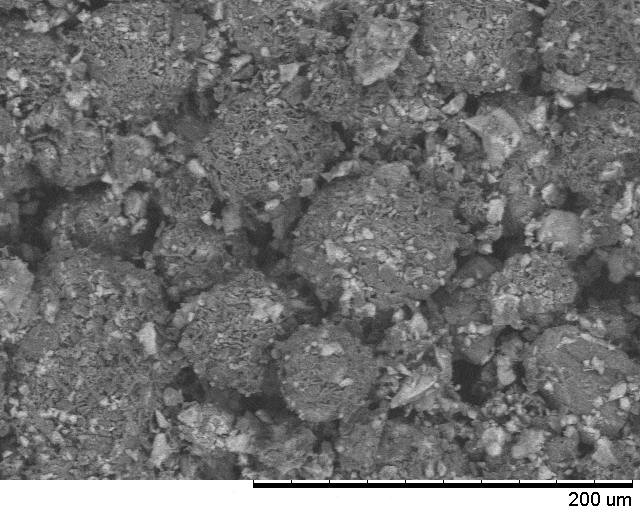

Supplement: Supplementary file 1 [file molecules-30-04469-s001.zip › SEM/Figure 1/sample with a NiFe ratio of 5-15_1.tif]

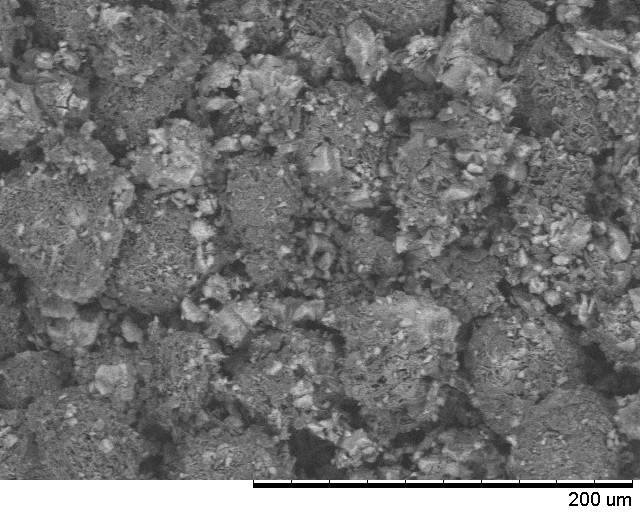

Supplement: Supplementary file 1 [file molecules-30-04469-s001.zip › SEM/Figure 1/sample with a NiFe ratio of 5-15_2.tif]

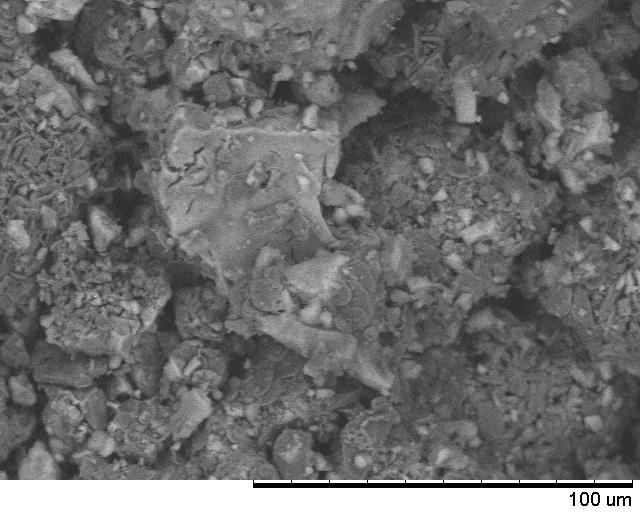

Supplement: Supplementary file 1 [file molecules-30-04469-s001.zip › SEM/Figure 1/sample with a NiFe ratio of 5-15_3.tif]

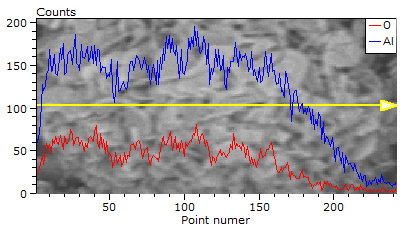

Supplement: Supplementary file 1 [file molecules-30-04469-s001.zip › SEM/Figure 2/prepared Al2O3 support_ Line1.tif]

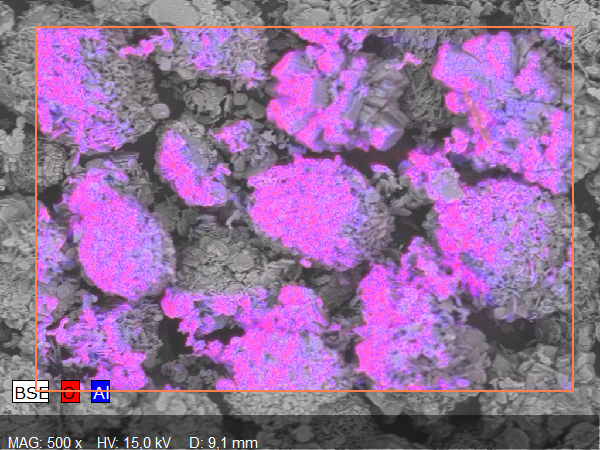

Supplement: Supplementary file 1 [file molecules-30-04469-s001.zip › SEM/Figure 2/prepared Al2O3 support_1.tif]

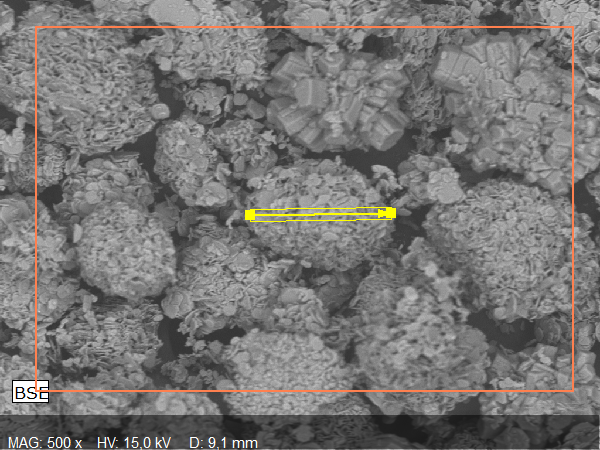

Supplement: Supplementary file 1 [file molecules-30-04469-s001.zip › SEM/Figure 2/prepared Al2O3 support_Line.tif]

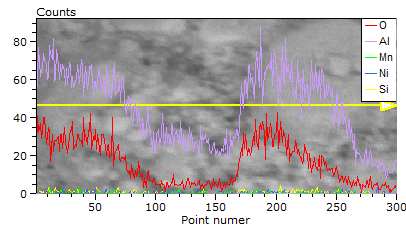

Supplement: Supplementary file 1 [file molecules-30-04469-s001.zip › SEM/Figure 2/sample with a NiFe ratio of 1-1_ Line.tif]

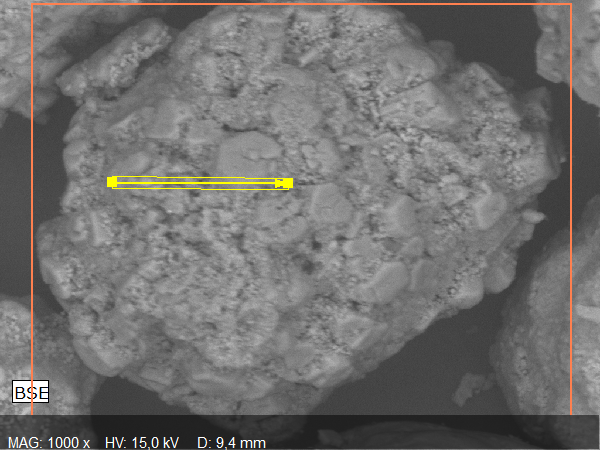

Supplement: Supplementary file 1 [file molecules-30-04469-s001.zip › SEM/Figure 2/sample with a NiFe ratio of 1-1_2Line1.tif]

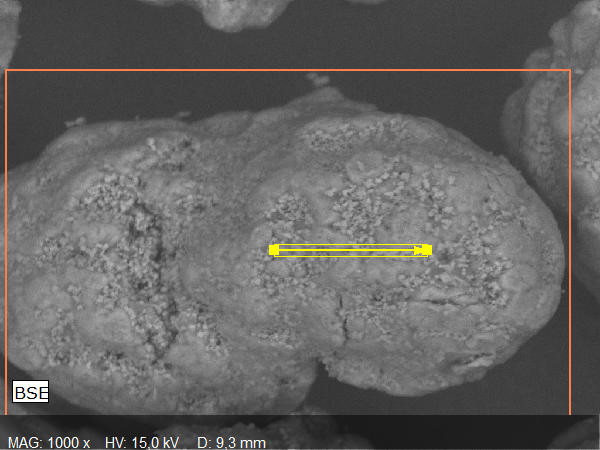

Supplement: Supplementary file 1 [file molecules-30-04469-s001.zip › SEM/Figure 2/sample with a NiFe ratio of 1-1_2_Line.tif]

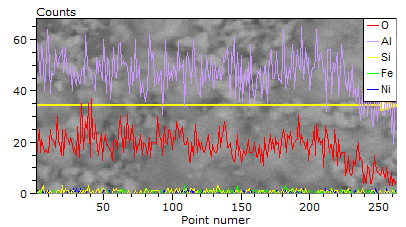

Supplement: Supplementary file 1 [file molecules-30-04469-s001.zip › SEM/Figure 2/sample with a NiFe ratio of 1-1_2_Line1.tif]

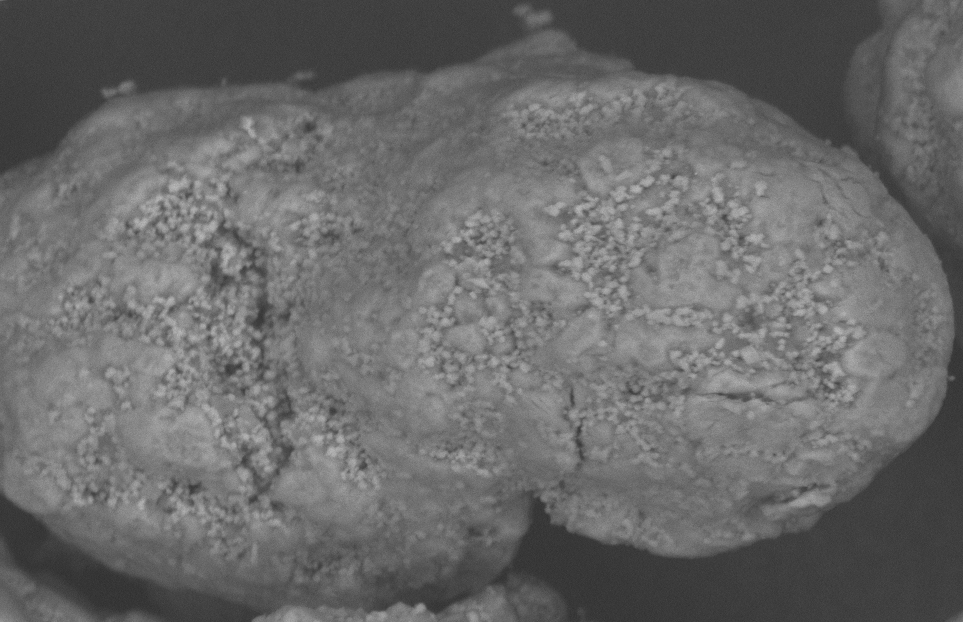

Supplement: Supplementary file 1 [file molecules-30-04469-s001.zip › SEM/Figure 2/sample with a NiFe ratio of 1-1_2_Map_BSE.tif]

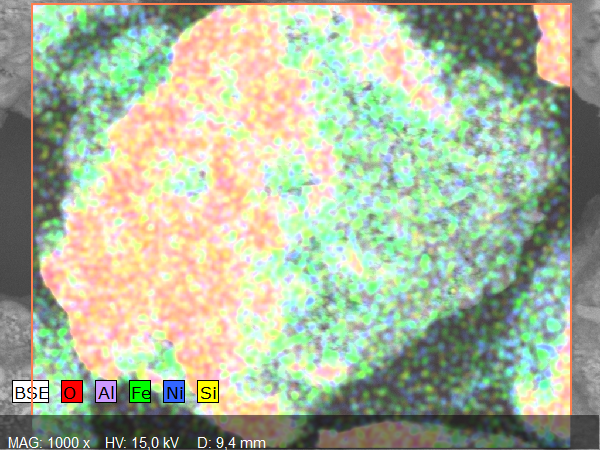

Supplement: Supplementary file 1 [file molecules-30-04469-s001.zip › SEM/Figure 2/sample with a NiFe ratio of 1-1_Map.tif]

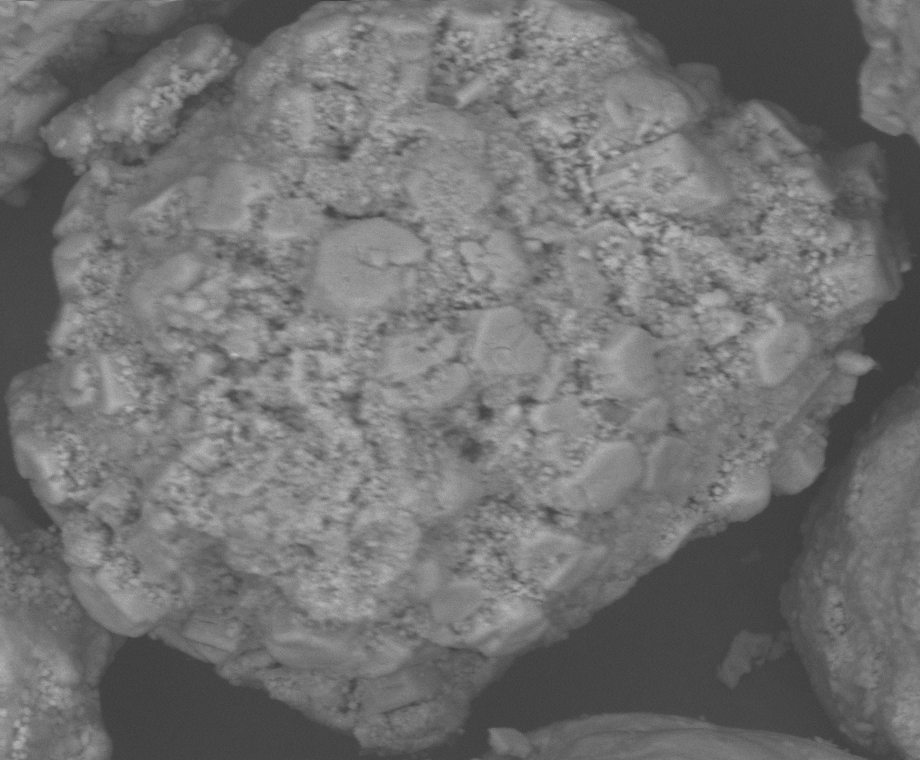

Supplement: Supplementary file 1 [file molecules-30-04469-s001.zip › SEM/Figure 2/sample with a NiFe ratio of 1-1_Map_BSE.tif]

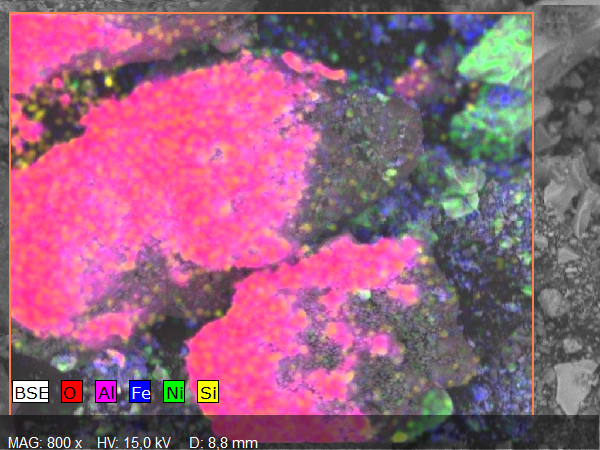

Supplement: Supplementary file 1 [file molecules-30-04469-s001.zip › SEM/Figure 2/sample with a NiFe ratio of 1-20_Map.tif]

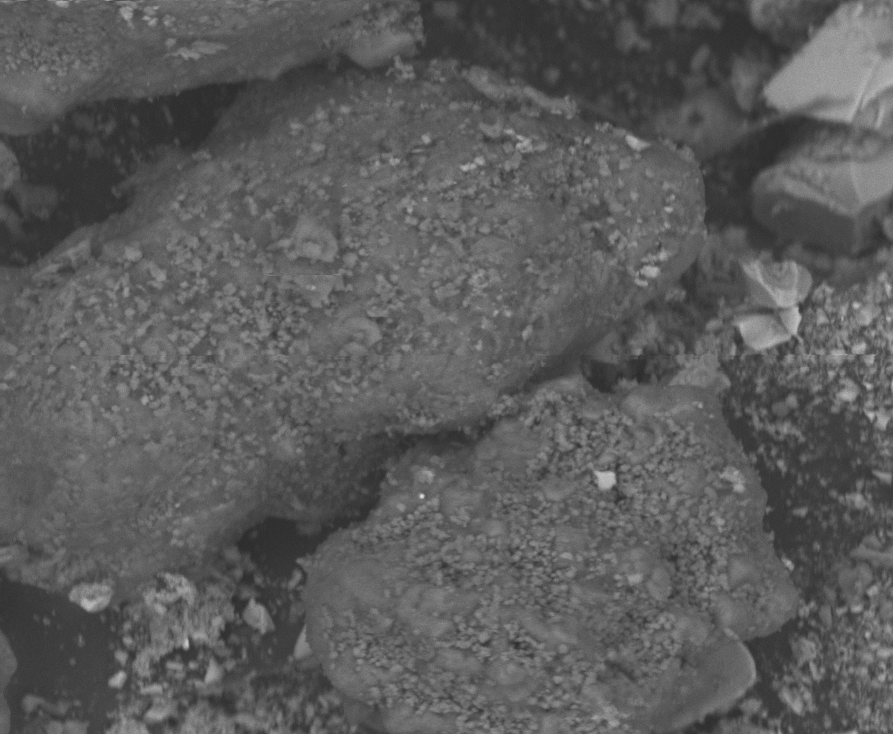

Supplement: Supplementary file 1 [file molecules-30-04469-s001.zip › SEM/Figure 2/sample with a NiFe ratio of 1-20_Map_BSE.tif]

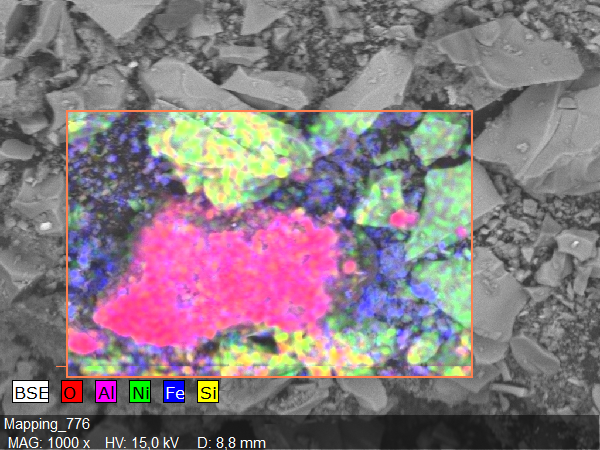

Supplement: Supplementary file 1 [file molecules-30-04469-s001.zip › SEM/Figure 3/sample with a NiFe ratio of 15-5_Map.tif]

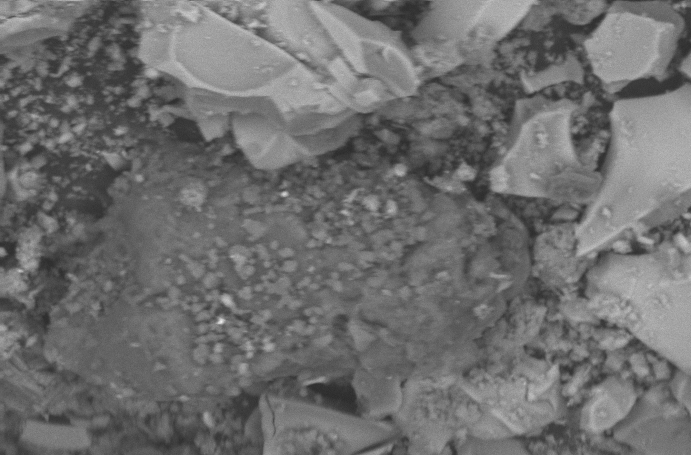

Supplement: Supplementary file 1 [file molecules-30-04469-s001.zip › SEM/Figure 3/sample with a NiFe ratio of 15-5_Map_BSE.tif]

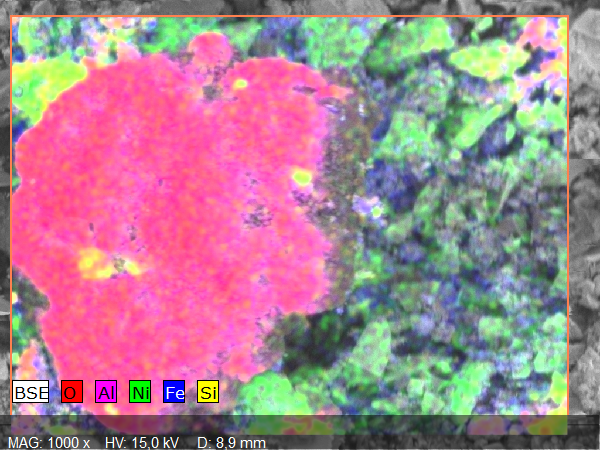

Supplement: Supplementary file 1 [file molecules-30-04469-s001.zip › SEM/Figure 3/sample with a NiFe ratio of 20-1_Map.tif]

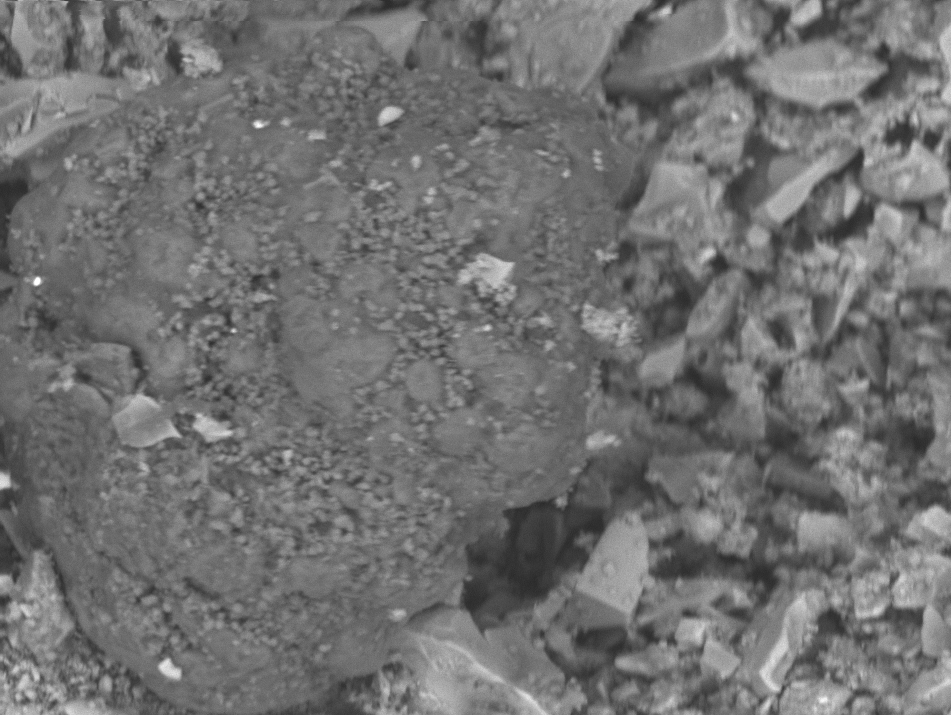

Supplement: Supplementary file 1 [file molecules-30-04469-s001.zip › SEM/Figure 3/sample with a NiFe ratio of 20-1_Map_BSE.tif]

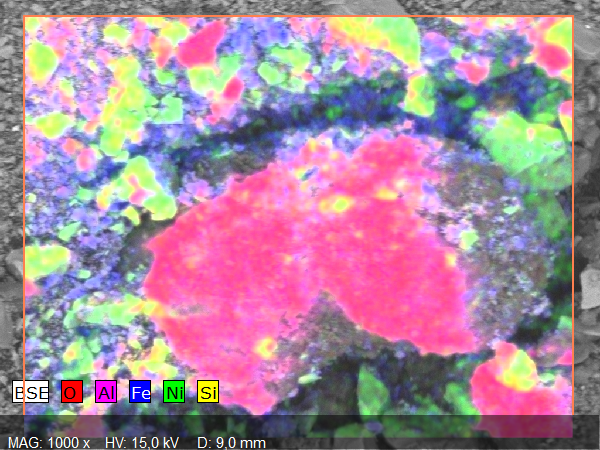

Supplement: Supplementary file 1 [file molecules-30-04469-s001.zip › SEM/Figure 3/sample with a NiFe ratio of 5-15_Map.tif]

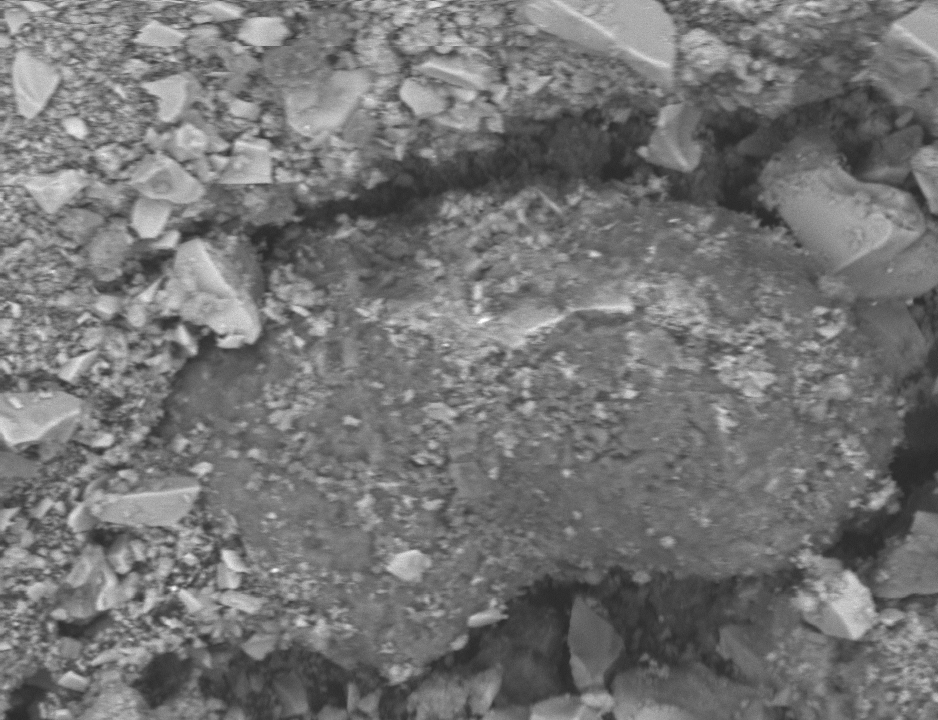

Supplement: Supplementary file 1 [file molecules-30-04469-s001.zip › SEM/Figure 3/sample with a NiFe ratio of 5-15_Map_BSE.tif]

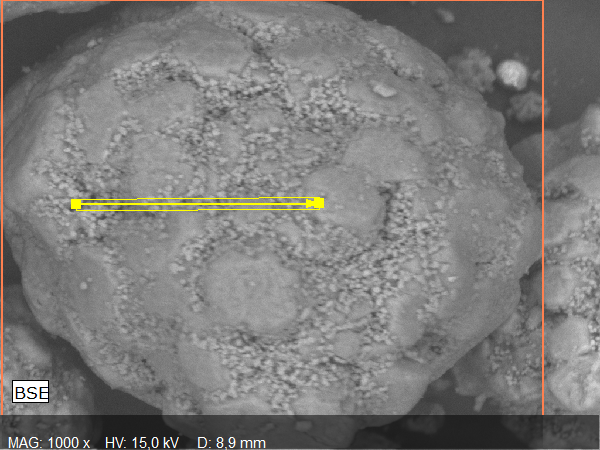

Supplement: Supplementary file 1 [file molecules-30-04469-s001.zip › SEM/Figure 5_5 degree C in minute/Line.tif]

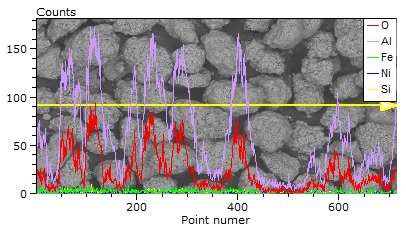

Supplement: Supplementary file 1 [file molecules-30-04469-s001.zip › SEM/Figure 5_5 degree C in minute/Line1.tif]

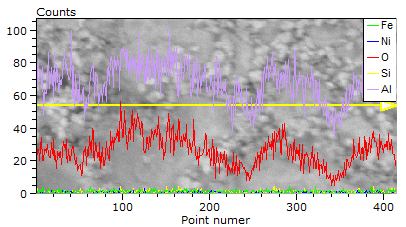

Supplement: Supplementary file 1 [file molecules-30-04469-s001.zip › SEM/Figure 5_5 degree C in minute/Line2.tif]

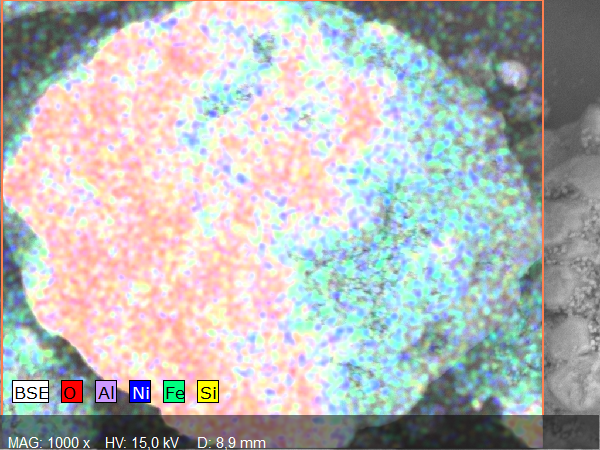

Supplement: Supplementary file 1 [file molecules-30-04469-s001.zip › SEM/Figure 5_5 degree C in minute/Map.tif]

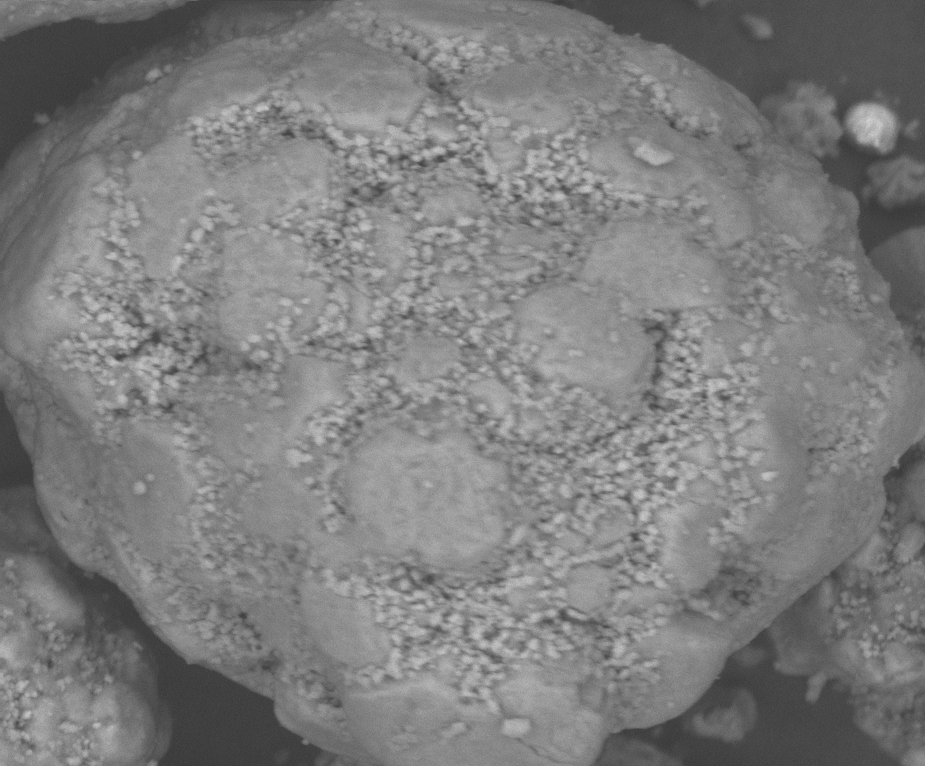

Supplement: Supplementary file 1 [file molecules-30-04469-s001.zip › SEM/Figure 5_5 degree C in minute/Map_BSE.tif]

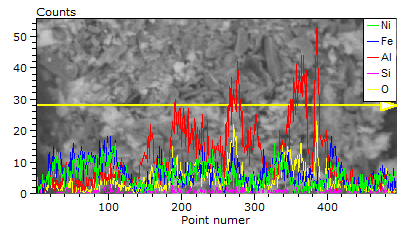

Supplement: Supplementary file 1 [file molecules-30-04469-s001.zip › SEM/Figure 6_6 degree C in minute/Line.tif]

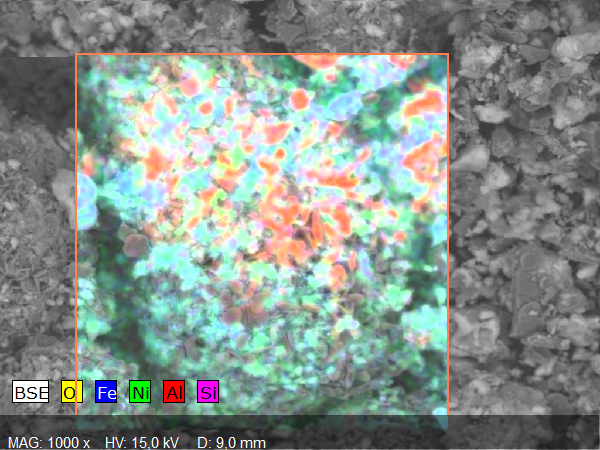

Supplement: Supplementary file 1 [file molecules-30-04469-s001.zip › SEM/Figure 6_6 degree C in minute/Map.tif]

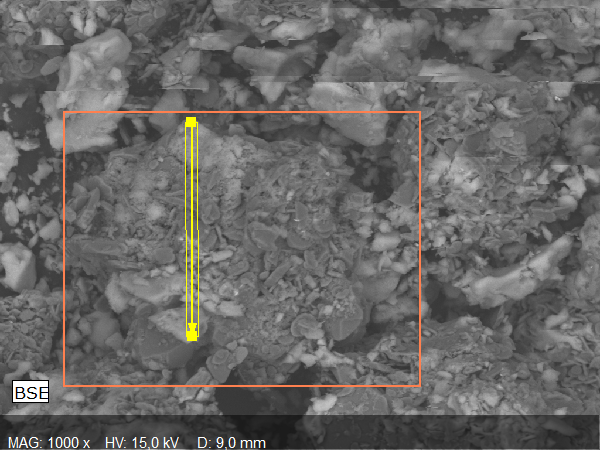

Supplement: Supplementary file 1 [file molecules-30-04469-s001.zip › SEM/Figure 7_10 degree C in minute/Line.tif]

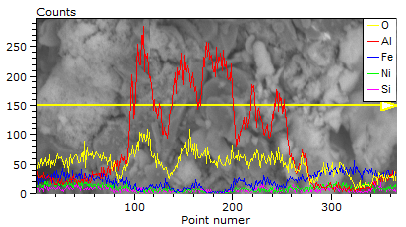

Supplement: Supplementary file 1 [file molecules-30-04469-s001.zip › SEM/Figure 7_10 degree C in minute/Line2.tif]

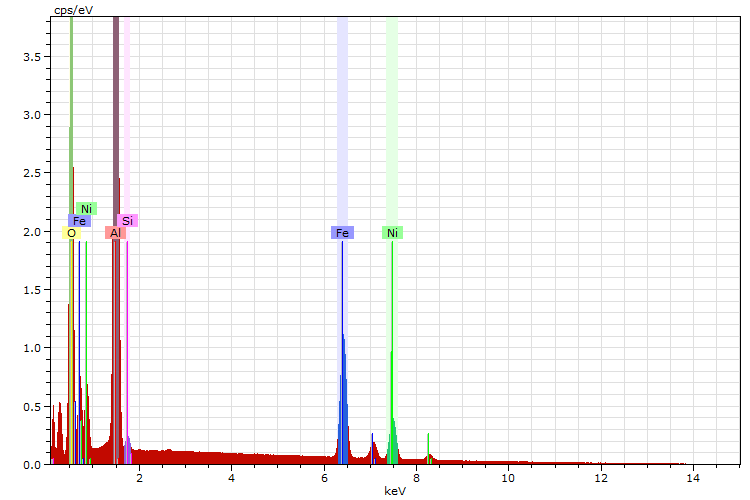

Supplement: Supplementary file 1 [file molecules-30-04469-s001.zip › SEM/Figure 7_10 degree C in minute/Map.tif]

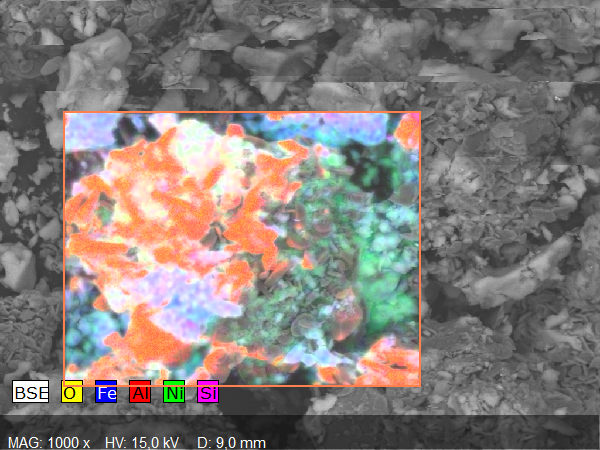

Supplement: Supplementary file 1 [file molecules-30-04469-s001.zip › SEM/Figure 7_10 degree C in minute/Map1.tif]

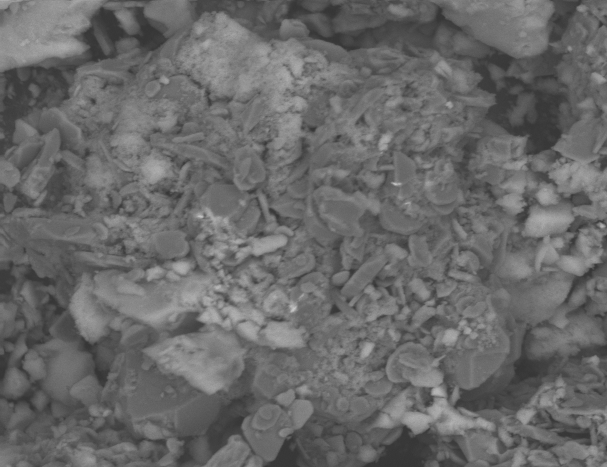

Supplement: Supplementary file 1 [file molecules-30-04469-s001.zip › SEM/Figure 7_10 degree C in minute/Map_BSE.tif]

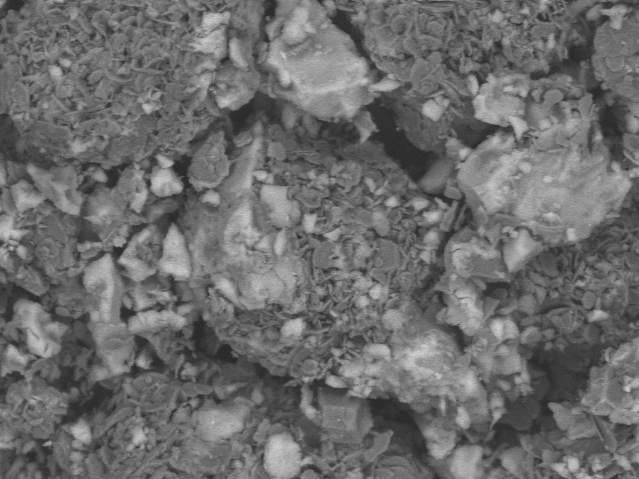

Supplement: Supplementary file 1 [file molecules-30-04469-s001.zip › SEM/Figure 7_10 degree C in minute/TM3000_4149(x1,0k).tif]

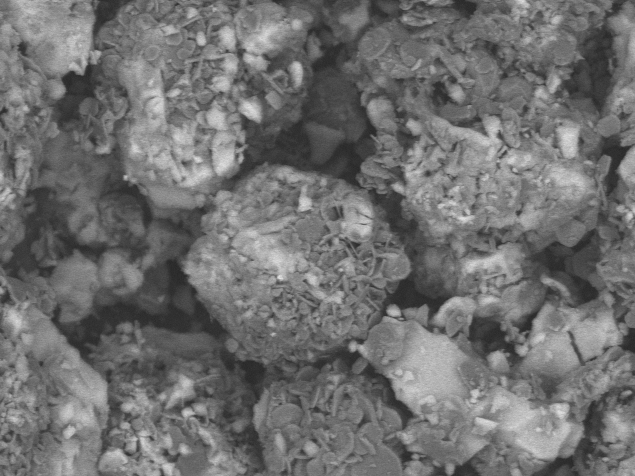

Supplement: Supplementary file 1 [file molecules-30-04469-s001.zip › SEM/Figure 7_10 degree C in minute/TM3000_4150(x1,0k).tif]
